# Chronic Sleep Disturbance Enhances Inflammation and Collagen Production in Neural- and Myofascial Tissues in Uninjured Rats

**DOI:** 10.3390/ijms27146106

**Published:** 2026-07-08

**Authors:** Mikhail A. Kolpakov, Betsy A. Kalicharan, Lewis Bright-Rowe, Frank L. Chen, Khyleisha A. Caesar, Yasmine B. Dahleh, Abby Kegg, Brendan A. Hilliard, Soul M. Moreno, Megan Van Der Bas, Parth R. Patel, Shrey Sitaram, Mary F. Barbe

**Affiliations:** 1 Aging + Cardiovascular Discovery Center, Department of Cardiovascular Sciences, Lewis Katz School of Medicine, Temple University, Philadelphia, PA 19140, USA; mikhail.kolpakov@temple.edu (M.A.K.); frank.chen0001@temple.edu (F.L.C.); yasmine.dahleh@temple.edu (Y.B.D.); brendan.hilliard@temple.edu (B.A.H.); soulysoulm@gmail.com (S.M.M.); shreysitaram@temple.edu (S.S.); 2 Biomedical Science Graduate Program, Lewis Katz School of Medicine, Temple University, Philadelphia, PA 19140, USA; megan.vanderbas@temple.edu (M.V.D.B.); ppatel2015@temple.edu (P.R.P.)

**Keywords:** sleep disturbance, pain, inflammation, fibrosis, musculoskeletal tissues, nerves

## Abstract

Chronic sleep disturbance is postulated to enhance pain and inflammatory responses, although systemic inflammation has been primarily investigated to date. We sought to examine the effects of chronic intermittent sleep disturbance on pain-related behaviors and peripheral forelimb neural- and myofascial tissues in the context of aging. Uninjured young adult and mature female rats (3 mo. and 9–10 mo. of age) were exposed to either normal or disturbed sleep for 9 h/day on 4 d/week (using in-cage environmental stimulation), for 6 weeks. Compared to rats with normal sleep, sleep disturbed rats of both ages displayed: (1) mild declines in grip strength; (2) low-grade elevations in immune cells and collagen in forepaw neural- and myofascial tissues; and (3) higher protein expression of collagen type I, CTGF, and phosphorylated AKT in forearm flexor muscles. Young adult sleep disturbed also showed increased TGF-beta. Mature sleep disturbed rats also displayed low-grade yet greatest forepaw mechanical sensitivity, muscle and serum TNF-α, and peripheral immune cell numbers. These data suggest that chronic intermittent sleep disruption can enhance pain-related behaviors, inflammation, and fibrosis-related changes in peripheral neural- and myofascial tissues in uninjured rats. These effects occurred in both age groups, yet were more pronounced in the older rats.

## 1. Introduction

Poor-quality or insufficient sleep commonly co-occurs with acute pain [1], painful flare-ups [2], and persistent pain [3]. Growing evidence suggests that insufficient sleep and pain can be bi-directional [4,5,6,7], with insufficient sleep leading to pain [6,8,9,10,11,12] and pain contributing to poor sleep [6,12]. Poor-quality sleep is also associated with enhanced inflammatory responses [13,14,15,16,17,18,19,20,21,22,23] that also appear to be bi-directional [17,20,24]. Individuals with infections or chronic conditions (including muscular dystrophies, diabetic neuropathies, and connective tissue disease-associated interstitial lung disease) frequently experience both increased inflammation and poor sleep [17,25,26,27,28,29], with many of these studies concluding that the chronic condition drove the sleep disturbance. Yet insufficient sleep has also been shown to activate inflammatory pathways that can increase the risks for infectious diseases, multi-organ injury, neuropathies, and metabolic diseases [14,18,22,25].

In humans, both acute and chronic sleep disturbance can lower pain thresholds [11,23,30,31], in parallel with increases in circulating pro-inflammatory cytokines [13,15,18,19,32] that can sensitize sensory nerves [33]. Only a few studies have examined the effects of chronic intermittent sleep disturbance on indices of inflammation in peripheral tissues of uninjured healthy individuals [16,23,30,31]. In one study with 24 healthy adults (8 males), prolonged partial sleep deprivation led to increased spontaneous production of interleukin-6 (IL-6) and tumor necrosis factor (TNF) by peripheral blood mononuclear cells [16]. In another, a 19-day in-hospital experimental model of prolonged disrupted and shortened sleep in healthy adult humans found reduced IL-6 production by monocytes [and increased plasma levels of IL-6 and C-reactive protein], presumably due to intermittent increases in cortisol [23]. Potential changes in immune cells in peripheral tissues were not examined in these studies.

In experimental animal studies, acute complete sleep deprivation results in increased circulating pro-inflammatory cytokines [34,35,36], particularly after injury [19,35]. Acute sleep fragmentation in rodents increases gene expression of inflammatory cytokines and transforming growth factor beta (TGF-β) in peripheral tissues (liver, spleen, heart) [36,37]. A study examining the effects of prolonged nearly complete sleep deprivation in mice for four days (mice were kept awake 96% of both their sleep and awake cycles), found profound increases in circulating cytokines and neutrophil numbers, and a high mortality rate (80%) [38]. We developed a wakefulness type stimulation method that included presentation of novel objects, cage and environmental manipulation, and sensory stimulation to animals in rat housing boxes [6,39], which we used to disturb the sleep of rats with overuse musculoskeletal injuries for 4 weeks, for 9–12 h/day, on 4 random days per week, post-injury. The rats in this study displayed high levels of several inflammatory and neuroimmune serum biomarker levels that correlated strongly with pain-related behaviors (yet no increase in serum corticosterone). The effect of this type of sleep disturbance on uninjured animals was not examined in these studies.

Thus, our objective here was to characterize the effects of prolonged intermittent sleep disturbance in uninjured rats on pain-related behaviors and inflammatory responses in forepaw/forelimb neural- and myofascial tissues, in the context of aging, since aging has been shown to increase inflammatory responses in a process that has been termed “inflammaging” [40,41]. For this, we compared the effects of 6 weeks of chronic intermittent sleep disturbance (9 h/day from 0700 to 1500, on 4 random days per week) versus normal sleep, in uninjured animals that were either young adult or mature in age (3 mo. versus 9 mo. of age at onset). We hypothesized that the sleep disturbance would enhance pain behaviors and inflammation (although we expected only low-grade responses since these were uninjured animals), and that aging would exacerbate these responses. Since we and others have previously established that inflammation can drive fibrosis-related tissue changes [42,43,44,45], we also sought to determine whether collagen deposition or production was altered in forearm neural- and myofascial tissues.

## 2. Results

### 2.1. Design, Body Weight, and Neural- and Myofascial Regions Examined in the Forepaw

**Figure 1:**
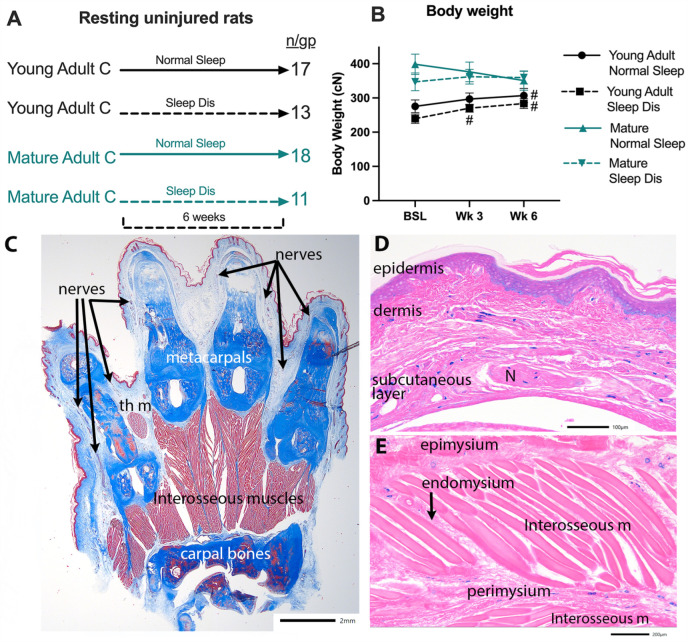
Design, body weight, and forepaw tissues examined. (**A**) Experimental Design, with numbers (n) of rats per age and sleep group indicated. Solid lines indicate rats exposed to normal sleep. Dashed lines indicate rats that underwent 6 weeks of intermittent sleep disturbance (Sleep Dis). (**B**) Body weight across the weeks. # *p* < 0.05, compared to baseline (BSL). n = 1–16/group. (**C**–**E**) Low power images of forepaw regions and tissues examined for neural- and myofascial tissue changes, including nerves (N) in the subcutaneous layer, interosseus muscles (m) and their endomysium. Staining and scale bars: Panel (**C**) (Masson’s trichrome), scale bar is 2 mm; Panel (**D**) (toluidine blue and eosin), scale bar is 100 microns; Panel (**E**) (toluidine blue and eosin), scale bar is 200 microns. The small blue stained cells in panels (**D**,**E**) within the nerve, fascia and muscle are mast cells. Th m = thenar muscle.

Uninjured, healthy, young adult and mature female rats (3.5 mo. and 9.5 mo. of age at onset, respectively) were exposed to either normal or sleep disturbance on 4 random days/week, for 9 h/day, for 6 weeks (Figure 1A). Body weight was tracked as a measure of general health. A repeated-measures, mixed-effects model with the factors *sleep*, *time* and *age*, showed a significant *time* × *age* interaction (*p* = 0.003). Post hoc tests showed that young adult rats (in both sleep groups) continued to gain weight over time, while mature rats maintained their body weight (Figure 1B). There were no between-group differences in body weight at 6 weeks.

Forepaw/forearm neuro- and myofascial tissues were examined. Specifically, nerves and associated fascial tissues in forepaw subcutaneous regions (Figure 1C,D), interossei muscles (Figure 1C,E), and forelimb flexor digitorum muscles were examined.

### 2.2. Enhanced Forepaw Mechanical Sensitivity in Mature Sleep Disturbed Rats; Reduced Grip Strength in Sleep Disturbed Rats of Both Age Groups

We first sought to determine if there were pain-related behavioral consequences, since chronic sleep disturbance has been shown enhance such behaviors [6,8,9,10,11,12,46,47].

**Figure 2:**
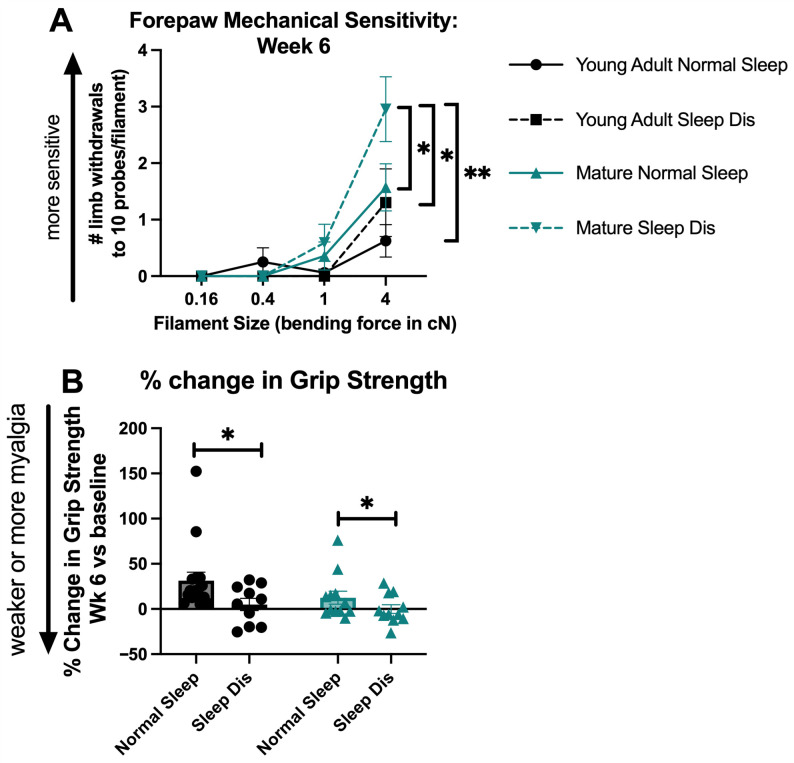
Sensorimotor behaviors. (**A**) Number of forelimb withdrawal responses to 10 monofilament probings per filament size, at 6 weeks. Higher numbers of limb withdrawals are indicative of mechanical sensitivity. (**B**) Percent change in reflexive grip strength in week 6 versus (vs.) baseline. Number (n) of animals analyzed per group: Young Adult Normal Sleep, n = 16; Young Adult Sleep Dis, 10; Mature Normal Sleep, n = 12–14; and Mature Sleep Dis, n = 11. * *p* < 0.05 and ** *p* < 0.01, compared between groups as shown.

At 6 weeks, forepaw sensitivity was analyzed using a three-way ANOVA with the factors, *monofilament size*, *age*, and *sleep*, and showed significant *monofilament size* × *age* and *monofilament size* × *sleep* interaction effects (*p* = 0.006 and *p* = 0.008, respectively). Post hoc analysis showed more forelimb withdrawal responses (interpreted as greater mechanical touch sensitivity) in Mature Sleep Dis rats in response to 4 cN monofilament probings, versus the other groups (*p* < 0.05 each; Figure 2A). The percent change in reflexive grip strength in week 6 versus baseline was analyzed using a two-way ANOVA with the factors *sleep* and *age* and showed a significant effect for *sleep* (*p* = 0.002). Post hoc analyses showed declines in both Sleep Dis groups, compared to the Normal Sleep groups (*p* = 0.049 each, Figure 2B).

### 2.3. There Was No Loss in Forelimb Flexor Cross-Sectional Area

One study in humans identified a negative association between trouble sleeping and a low muscle quality index (calculated as handgrip strength and appendicular skeletal muscle mass) [48], and another study observed an association between complete sleep deprivation and sarcopenia [49]. Therefore, we quantified the cross-sectional area of this muscle at it widest point. No significant differences between groups were observed (Figure S1). This finding suggests that flexor muscle loss was not contributing to the grip strength declines.

### 2.4. Serum TNF-α Levels Were Elevated in Mature Animals Undergoing 6-Weeks of Sleep Disturbance; Corticosterone Levels Were Not Altered

**Figure 3:**
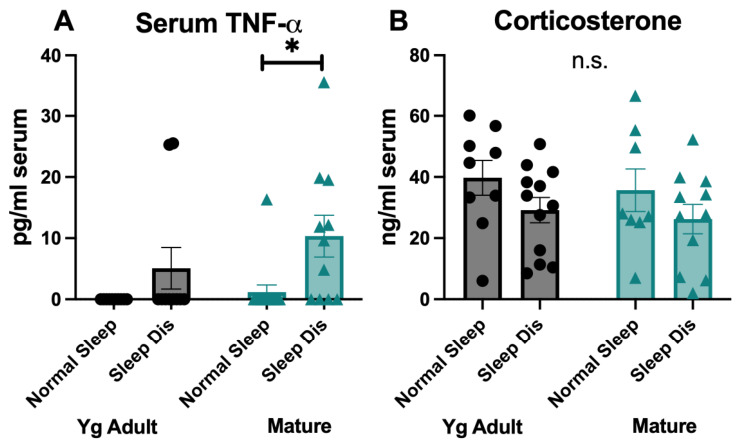
Serum levels of TNF-α and corticosterone. (**A**,**B**) Serum levels of TNF-α (n = 10–14/group) and corticosterone (n = 8–12/group), tested using ELISA. * *p* < 0.05, compared between groups as shown, n.s. = not significant.

Since sleep disturbance is often associated with increased serum levels of TNF-α [19,34,50], a potent pro-inflammatory cytokine [51], we examined serum for TNF-α using ELISA. A two-way ANOVA revealed a significant effect of *sleep* on serum TNF-α (*p* = 0.005). Post hoc analyses showed that only Mature Sleep Dis rats contained higher serum TNF-α, compared to Mature Normal Sleep rats (*p* = 0.006; Figure 3A), although two Young Adult Sleep Dis rats also showed high serum TNF-α.

Corticosterone levels did not differ between the groups (Figure 3B).

### 2.5. Sleep Disturbance Induced Low-Grade Increases in Immune Cells Within Nerves

**Figure 4:**
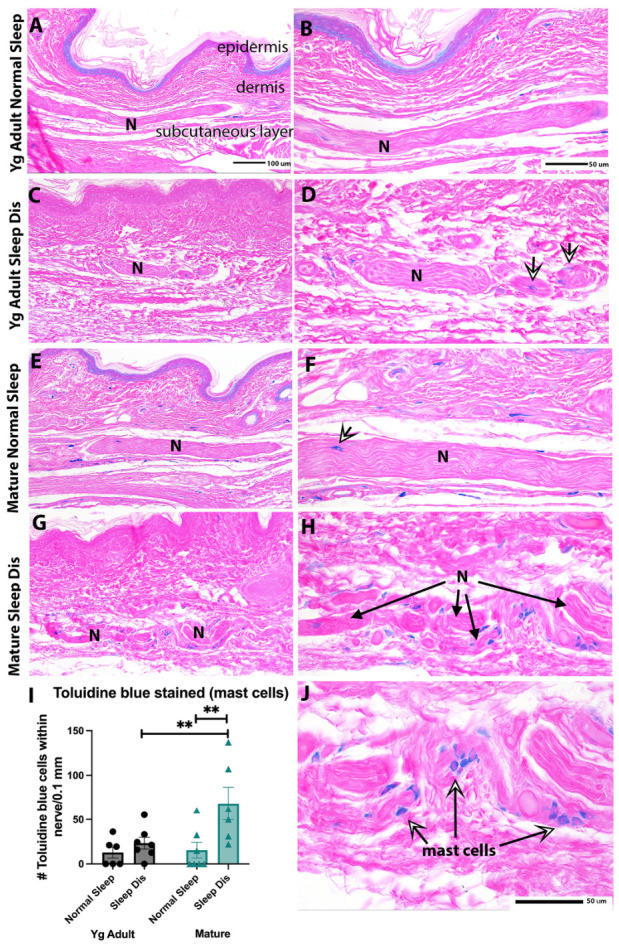
Toluidine blue stained mast cells in forepaw nerves. Examples of nerves are delineated by “N” and black arrows. Eosin counterstain. (**A**–**H**,**J**) Representative examples of blue stained mast cells in nerves (white arrows) in each group. Lower power images are on the left; higher power images are on the right. (**I**) Quantification of numbers of toluidine blue stained mast cells. Scale bar in panel (**A**) applies to (**C**,**E**,**G**); scale bar in (**B**) applies to (**D**,**F**,**H**). n = 6–7/group. ** *p* < 0.01, compared between groups as shown. N = nerve.

**Figure 5:**
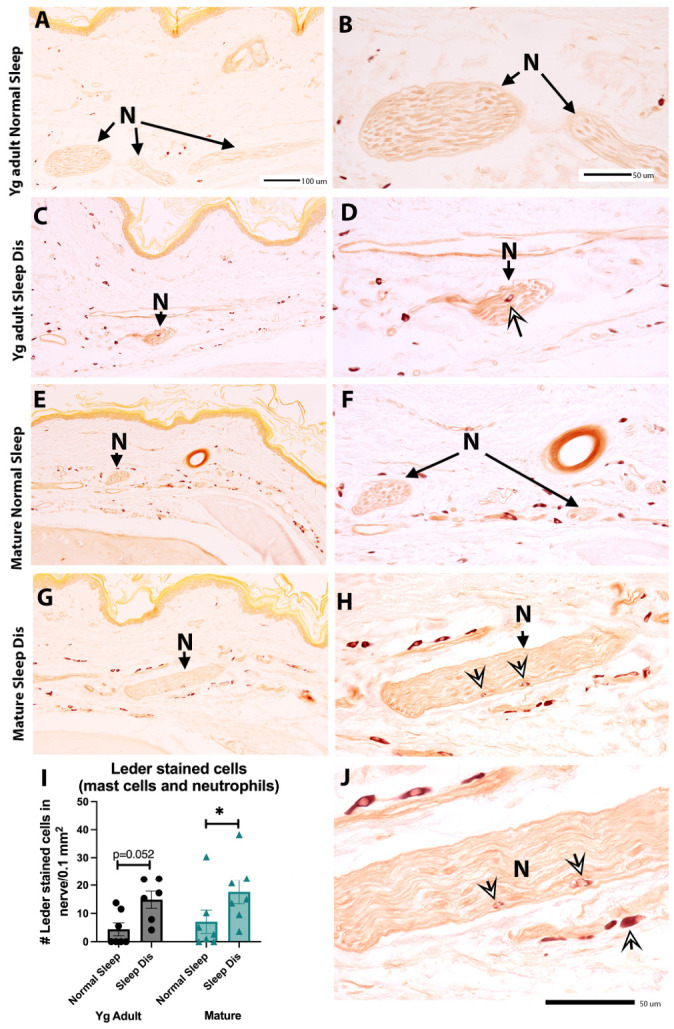
Leder stain detected mast cells and neutrophils within forepaw nerves. Examples of nerves are delineated by “N” and black arrows. (**A**–**H**,**J**) Representative images from each group, with lower power images on the left and higher power images on the right. Representative Leder stain detected cells are indicated with white arrows. (**I**) Quantification of Leder detected cells. Scale bar in panel (**A**) applies to (**C**,**E**,**G**); scale bar in (**B**) applies to (**D**,**F**,**H**). n = 6–7/group. * *p* < 0.05, compared between groups as shown. N = nerve.

**Figure 6:**
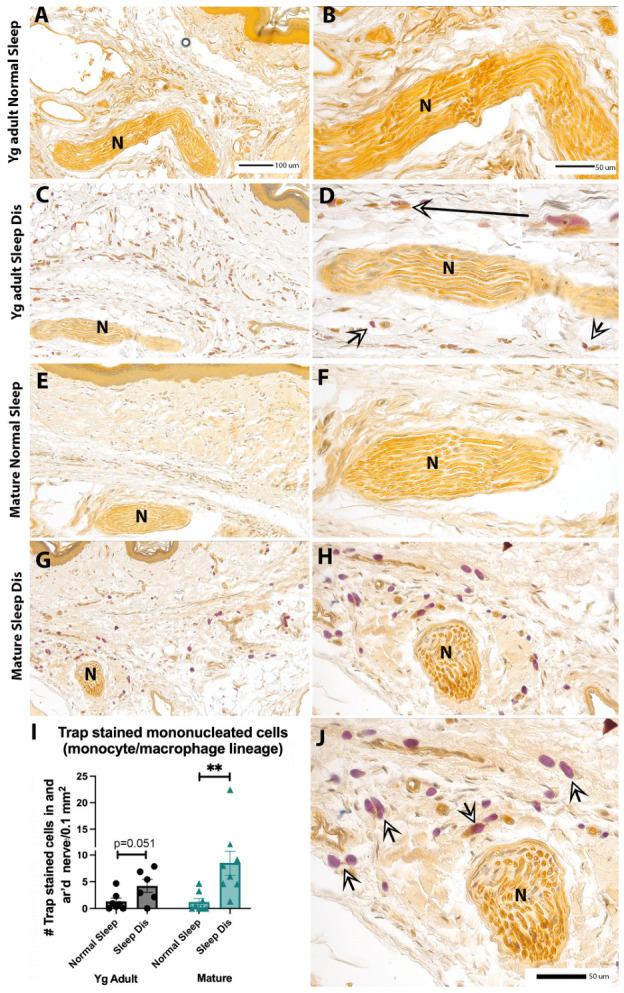
TRAP-stained mononucleated cells (macrophages) around forepaw nerves (examined to 20 microns external to the outer perineurium). (**A**–**H**,**J**) Representative images from each group, with lower power images on the left and higher power images on the right. (**I**) Quantification of TRAP-stained mononucleated cells. Scale bar in panel (**A**) applies to (**C**,**E**,**G**); scale bar in (**B**) applies to (**D**,**F**,**H**). n = 6–8/group. ** *p* < 0.01, compared between groups as shown. N = nerve.

Since sensorimotor behaviors can be influenced by inflammation in forepaw and forearm neuromuscular tissues [52,53,54,55], we next examined these tissues for potential inflammatory changes. We examined forepaw nerves in subcutaneous tissues (see Figure 1C,D) for presence of immune cells (toluidine blue detected mast cells [56,57], Leder stain detected mast cells and neutrophils [56,58], and TRAP stain detected mononucleated cells that are considered as macrophages in soft tissues [59,60,61]; Figure 4, Figure 5 and Figure 6, respectively).

Examination of toluidine blue stained tissues (Figure 4A–H,J) showed consistently more mast cells (stained blue; examples indicated by white arrows) in nerves of Mature Sleep Dis rats, relative to Mature Normal Sleep rats (Figure 4G,H vs. Figure 4E,F). A two-way ANOVA showed significant effects for both *sleep* and *age* for mast cell numbers within the nerves (*p* = 0.008 and *p* = 0.04, respectively). Post hoc analysis showed more toluidine blue detected mast cells in Mature Sleep Dis rats, compared to Mature Normal Sleep rats and Young Adult Sleep Dis rats (Figure 4I).

More Leder cells (dark brown stained mast cells and neutrophils) were visible in Sleep Dis rats of both age groups, relative to Normal Sleep rats (Figure 5A–H). A two-way ANOVA showed significant effects of *sleep* only (*p* = 0.007). A post hoc analysis showed that Leder-stained cells were increased in forepaw nerves of Mature Sleep Dis rats than Mature Normal Sleep rats (*p* = 0.04; Figure 5I). There was also a strong trend towards an increase in Young Adult Sleep Dis rats than Young Adult Normal Sleep rats (*p* = 0.052).

More TRAP-stained mononucleated cells (dark magenta colored), known to be macrophages in soft tissues [59,60,61], were observed in paraneural tissues (although not intra-neurally) of Mature Sleep Dis rats, versus Mature Normal Sleep rats (Figure 6A–H,J). A two-way ANOVA showed significant effects of *sleep* only for TRAP-stained cells around the nerves (*p* = 0.002). A post hoc analysis showed these cells were increased in forepaw nerves of Mature Sleep Dis rats, compared to Mature Normal Sleep rats (*p* = 0.001; Figure 6I). There was also a strong trend towards an increase in Young Adult Sleep Dis, compared to Young Adult Normal Sleep rats (*p* = 0.051).

### 2.6. Sleep Disturbance Induced Low-Grade Increases in Collagen Deposition in and Around Nerves

**Figure 7:**
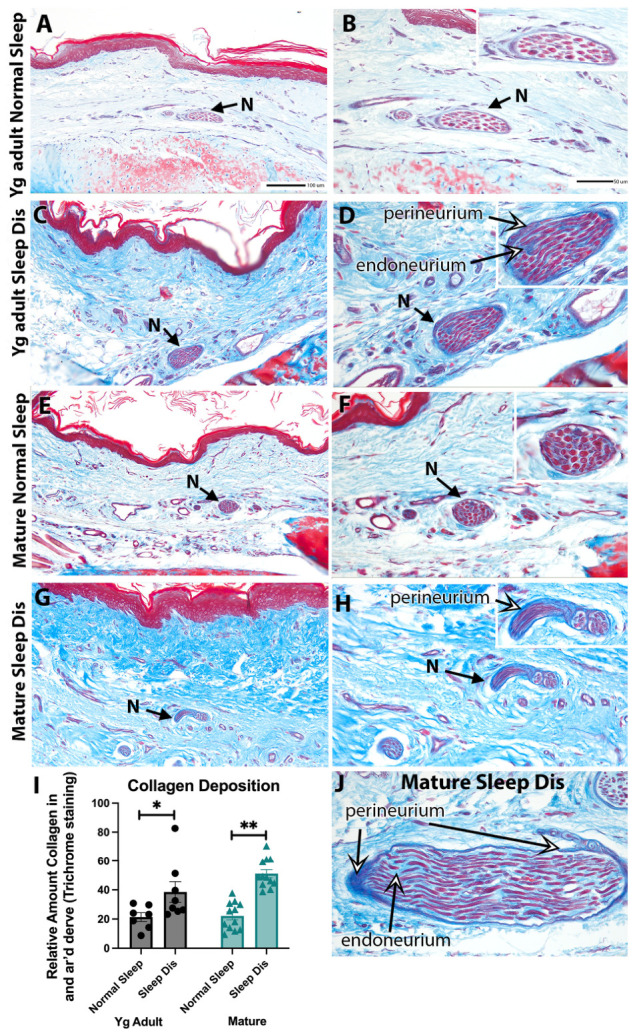
Collagen deposition in and around forepaw nerves (examined to 20 microns external to the outer perineurium). Examples of nerves are delineated by “N” and black arrows. (**A**–**H**) Representative images from each group, with lower power images on the left and higher power images on the right. Panel (**J**) shows a higher power Mature Sleep Dis rat’s nerve with increased collagen staining in the endoneurium and perineurium (examples indicated by white arrows) relative to Normal Sleep rats. (**I**) Quantification of collagen deposition within and around nerve profiles. Scale bar in panel (**A**) applies to (**C**,**E**,**G**); scale bar in (**B**) applies to (**D**,**F**,**H**,**J**). n = 7–12/group. * *p* < 0.05 and ** *p* < 0.01, compared between groups as shown. N = nerve.

Since inflammatory responses can contribute to fibrotic responses [42,43], we next examined collagen deposition in forepaw neural fascial tissues using Masson’s trichrome staining (which stains all collagen subtypes blue; Figure 7). We observed an increase in collagen deposition in peri- and endoneurium fascia layers of nerves of Sleep Dis rats (both age groups), relative to the Normal Sleep rats (Figure 7D,H,J). We quantified the amount of collagen deposition within and around the nerves. A two-way ANOVA showed a significant effect for *sleep* (*p* < 0.0001). Post hoc analyses showed that there was more collagen deposition (i.e., staining) within and around nerves in the forepaw subcutaneous tissues in Sleep Dis rats of both age groups, compared to Normal Sleep rats (*p* = 0.02 young adult, and *p* < 0.0001 mature, Figure 7I).

### 2.7. Sleep Disturbance Induced Low-Grade Increases in Immune Cells in Fascia of Forepaw Intrinsic Muscles

**Figure 8:**
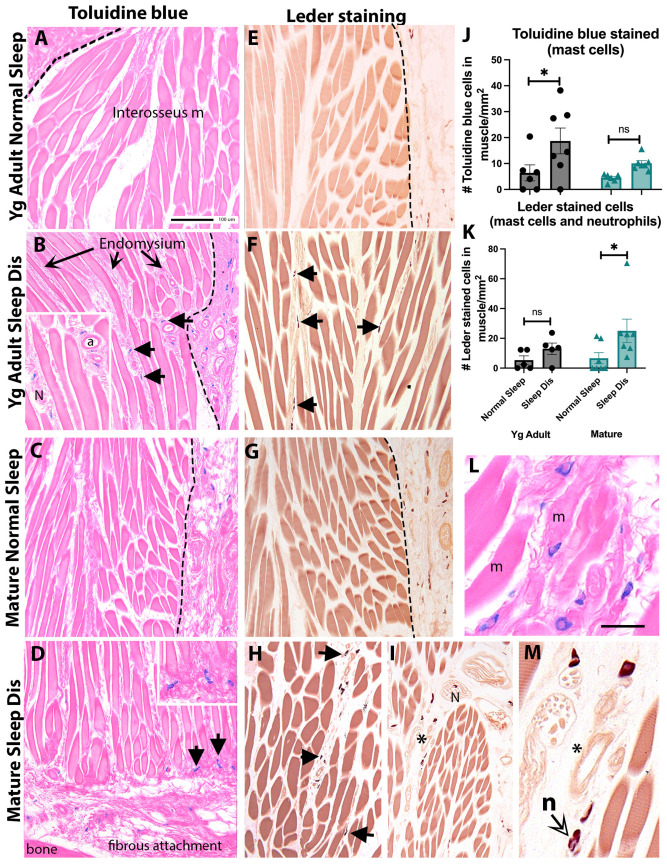
Representative images of toluidine blue stained mast cells, and Leder-stained mast cells and neutrophils, in forepaw intrinsic interossei muscles. Dashed line separates region counted (left side of panel) from external fascial layer (a region not included in the quantification) in panels (**A**–**C**,**H**). (**A**–**D**,**L**) Toluidine blue stained mast cells in muscles. a = artery. Eosin counterstain. (**E**–**I**,**M**) Leder-stained cells. (**J**) Quantification of Toluidine blue stained mast cells. (**K**) Quantification of Leder stain detected mast cells and neutrophils in forepaw intrinsic muscles. (**L**) Higher power images of toluidine blue stained cells around individual myofibers. (**M**) Higher power images of Leder-stained cells in the muscles indicated with an asterisk in panel I, with n and white arrow indicating a neutrophil shaped cell. Scale bar in panels A applies to panels (**B**–**I**). Scale bar in panel (**A**) applies to other images as well. n = 5–7/group. * *p* < 0.05, compared between groups as shown; ns = not significant.

We expanded our investigation to nearby forepaw myofascial tissues, focusing on interossei muscles and their endomysium (Figure 1C,E and Figure 8), since inflammatory responses in muscles can contribute to grip strength declines [62]. Interossei muscles of Sleep Dis rats showed more toluidine blue stained mast cells and Leder-stained mast cells and neutrophils in several locations within the intrinsic forepaw muscles of Sleep Dis rats regardless of age (Figure 8), relative to Normal Sleep rats. These cells were seen near nerves within the muscle tissue (Figure 8B), at fibrous attachment points to forepaw bones (Figure 8D), in the fascial septa penetrating the muscles (Figure 8F,H,I,M), and sometimes external to individual myofibers (Figure 8L). Although these increases were small, two-way ANOVAs revealed significant effects of *sleep* for mast cells (*p* = 0.009) and Leder-stained cells (*p* = 0.03). Post hoc analyses revealed increased mast cells in Young Adult Sleep Dis rats, compared to Young Adult Normal Sleep rats (*p* = 0.01, Figure 8J), and increased Leder-stained cells in Mature Sleep Dis rats, compared to Mature Normal Sleep rats (*p* = 0.02, Figure 8K).

We also examined the interossei muscles for TRAP-strained mononucleated cells (Figure S2), and observed a small increase, particularly in the Young Adult Sleep Dis group. A two-way ANOVA showed a small significant effect of *sleep* only (*p* = 0.049). A post hoc test showed a significant increase in Young Adult Sleep Dis rats, compared to Young Adult Normal Sleep rats (*p* = 0.048; Figure S2).

### 2.8. Sleep Disturbance Induced Increases in Collagen Deposition in Fascia of Forepaw Intrinsic Muscles

**Figure 9:**
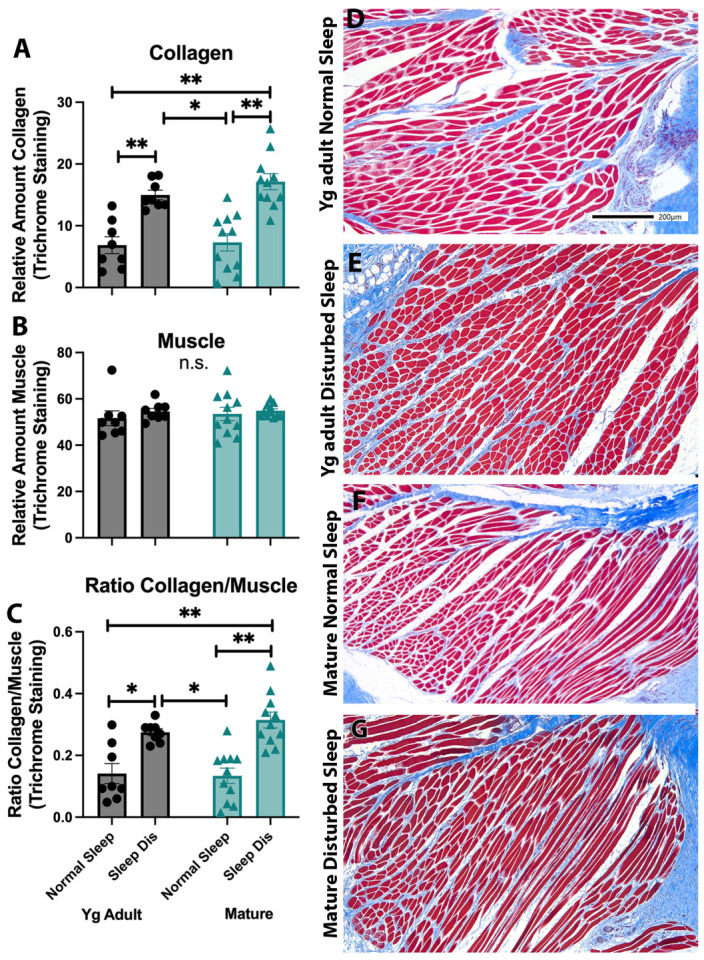
Relative amount of collagen and muscle in mid-forepaw intrinsic muscles, detected using Masson’s trichrome staining. (**A**) Relative amount collagen. (**B**) Relative amount of muscle. (**C**) Ratio of relative amounts of collagen versus muscle. n = 8–11/group. * *p* < 0.05 and ** *p* < 0.01, compared between groups as shown; n.s. = not significant. (**D**–**G**) Representative examples of staining of mid-forepaw intrinsic muscles in each group. Collagen stains blue and muscles stain red after Masson’s trichrome staining. Scale bar in (**D**) applies to each panel.

Since collagen deposition within forepaw muscles can contribute to grip strength declines [52,53], we quantified collagen deposition in intrinsic forepaw muscles. Representative images showed a clear increase in collagen deposition (blue staining) in the endomysium of interossei muscles of Young Adult and Mature Sleep Dis rats, relative to Normal Sleep rats (Figure 9A,D–G). A two-way ANOVA showed a significant effect for *sleep* (*p* < 0.0001). Post hoc analyses revealed that there was more collagen staining in Sleep Dis rats (regardless of age), compared to those with Normal Sleep (*p* < 0.05 or *p* < 0.01, as shown in Figure 9A). The relative amount of interossei muscle was not altered by sleep or age (Figure 9B). This finding suggests that loss of muscle in the forepaw was not contributing to the grip strength declines. The ratio of collagen to muscle showed a *sleep* effect (*p* < 0.0001), and post hoc analyses showed higher ratios in Sleep Dis rats, compared to Normal Sleep rats (Figure 9C).

### 2.9. Tendon Tissues Were Not Affected by Sleep Disturbance or Maturation

We examined intrinsic forepaw tendons and associated epitenon for any signs of histopathology. No significant histopathological changes were detected in any group, and there were no between-group differences (Figure S3).

### 2.10. Sleep Disturbance Induced Low-Grade Increases in CD68 Cells and TNF-α Immunoexpression in Forelimb Flexor Digitorum Muscles

**Figure 10:**
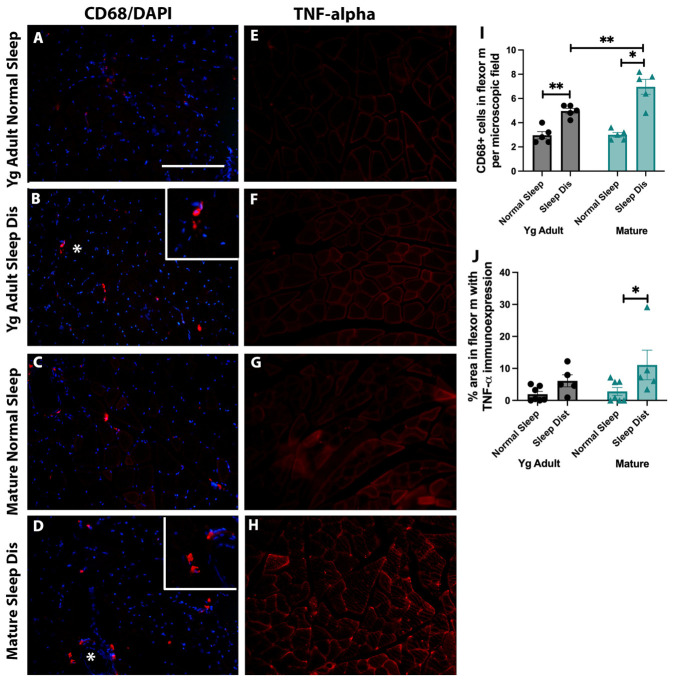
Immunohistochemically detected CD68-immunopositive cells and TNF-α immunoexpression in cross-sectionally cut forelimb flexor digitorum muscle. (**A**–**D**) Immunohistochemically detected CD68-immunopositive cells (red cells). DAPI (blue) co-staining also shown. Insets in (**B**,**D**) are higher power images of the red CD68+ cells indicated with asterisks (*). (**I**) Quantification of numbers of CD68-immunopositive cells. (**E**–**H**) Representative images of TNF-α immunoexpression. (**J**) Percent area of TNF-α immunoexpression within the muscle. Scale bar in (**A**) applies to the other main panels and is 100 microns. n = 5/group. * *p* < 0.05 and ** *p* < 0.01, compared between groups as shown.

We next expanded our examinations to forelimb flexor digitorum muscles to determine if they also showed an increase in immune cells. Although small in number, there were more CD68-immunopositive cells in the flexor muscles of Sleep Dis rats (regardless of age), relative to Normal Sleep rats (Figure 10A–D). After quantification, a two-way ANOVA showed a *sleep* × *age* interaction effect (*p* = 0.02). Post hoc analyses showed increased numbers of CD68+ cells in Sleep Dis rats regardless of age, compared to those with Normal Sleep (*p* = 0.008 for Young Adult and *p* < 0.0001 for Mature), and in Mature Sleep Dis rats compared to younger Sleep Dis rats (*p* = 0.008) (Figure 10I).

TNF-α immunoexpression was higher only in the Mature Sleep Dis rats (Figure 10H vs. Figure 10E–G). After quantification, a two-way ANOVA showed a *sleep* effect (*p* = 0.01). Post hoc analyses showed increased TNF-α immunoexpression in the flexor muscles of Mature Sleep Dis rats, compared to Mature Normal Sleep rats (*p* = 0.03, Figure 10J).

### 2.11. Sleep Disturbance Induced Low-Grade Increases in Collagen Type I and CTGF in Forelimb Flexor Digitorum Muscles

**Figure 11:**
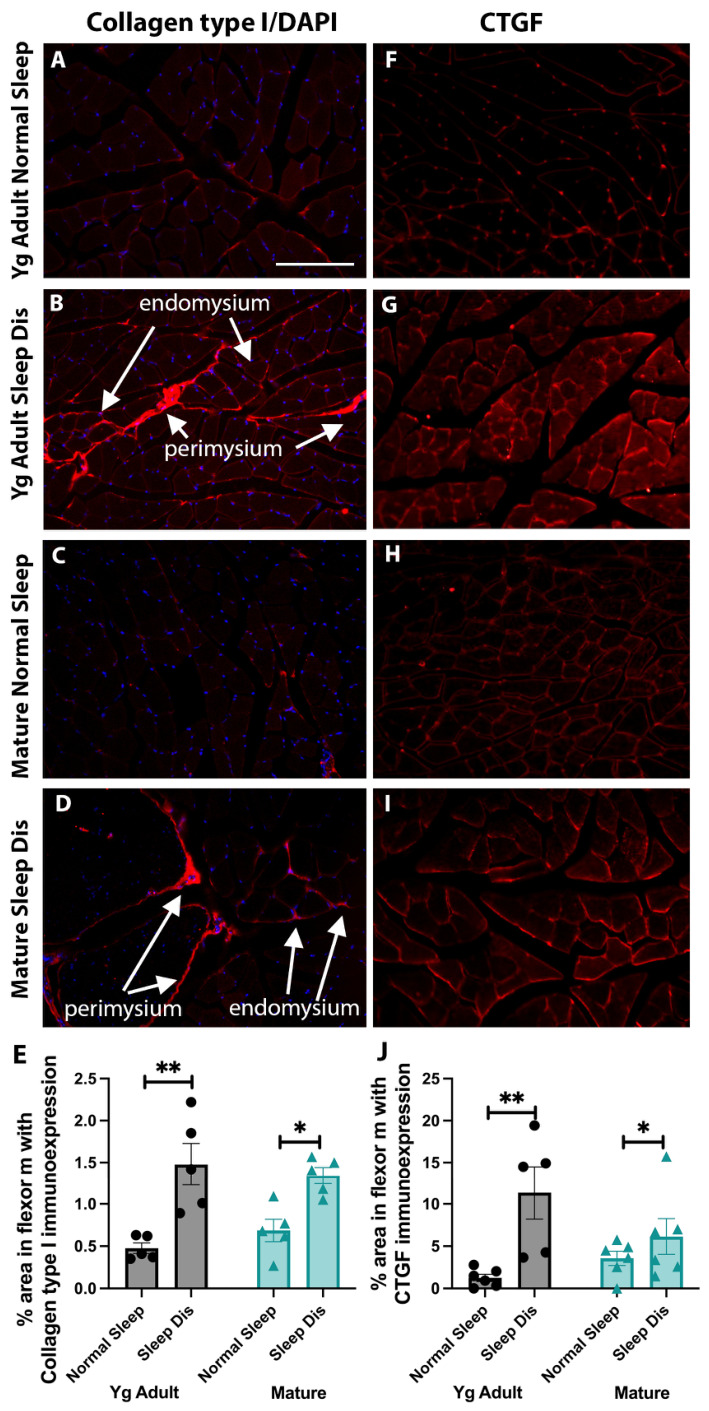
Immunohistochemically detected collagen type I and CTGF in cross-sectionally cut forelimb flexor digitorum muscle. (**A**–**D**) Immunohistochemically detected collagen type I (red in color) in perimysium (around muscle fascicles) and endomysium structures (around individual myofibers; white arrows). DAPI counterstain (blue). (**E**–**H**) Immunohistochemically detected CTGF (red in color) around individual myofibers. (**I**) Quantification of percent area with collagen immunoexpression within the muscle. (**J**) Quantification of percent area with CTGF immunoexpression within the muscle. Scale bar in A applies to other images and is 100 microns. n = 5/group. * *p* < 0.05 and ** *p* < 0.01, compared between groups as shown.

The forelimb flexor digitorum muscles were also examined for immunoexpression of collagen type I and connective tissue growth factor (CTGF) (Figure 11). Representative images show an increase in collagen type I immunoexpression (red color) in the endo- and perimysium of these muscles in Young Adult and Mature Sleep Dis rats, relative to Normal Sleep rats (Figure 11A–D). CTGF immunoexpression was visible in small cells at the perimeters of individual myofibers in Normal Sleep rats (both ages), yet as increased deposition in the endomysium of Sleep Dis rats (both ages) (Figure 11F–I). Two-way ANOVAs showed significant effect of *sleep* for collagen type I and CTGF immunoexpression (*p* < 0.0001 and *p* = 0.003), respectively). Post hoc analyses showed more expression of each in Sleep Dis rats, regardless of age, compared to Normal Sleep rats (collagen type I: *p* = 0.006 for Young Adult, *p* = 0.02 for Mature, Figure 11E; CTGF: *p* = 0.006 for Young Adult, *p* = 0.049 for Mature, Figure 11J).

### 2.12. Sleep Disturbance Induced Changes in Select Muscle Proteins

**Figure 12:**
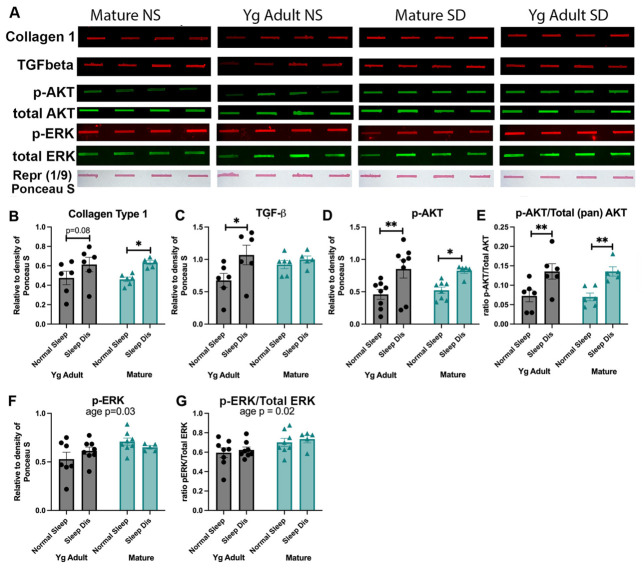
Examination of collagen type 1, TGF-β, p-AKT, Total AKT, p-ERK, and total ERK in homogenized samples of forelimb flexor digitorum muscles via slot blot. Full images of slot blots and all replications are shown in Figures S4 and S5. (**A**) Representative slot blot membranes in which 4 of the 5–8 different samples loaded for each group are shown. These membranes were probed with primary antibodies as labeled. Ponceau S staining of one of nine representative membrane (from the TGF-β probed membrane) is shown below. The other eight Ponceau S stained membranes can be found in Supplemental Figures S4 and S5. (**B**–**D**,**F**) Quantification of the density each band relative to the Ponceau S staining of the same band, with n = 5 to 8/group is shown for collagen type 1, TGF-β, p-AKT, and p-ERK. (**E**,**G**) Ratios of p-AKT to total (pan) AKT, and p-ERK to total ERK, are shown. * *p* < 0.05 and ** *p* < 0.01, compared between groups as shown except for p-ERK and p-ERK/total ERK in which there were no post hoc findings (only the main effect for *age* is listed).

To identify other muscle proteins that might be responding to the sleep disturbance, we examined the subset of flexor forelimb muscles that had been homogenized for changes in collagen type 1, TGF-β, p-AKT, total AKT, p-ERK and total ERK using slot blot methodology. Representative slot blot bands are shown in Figure 12A, and whole slot blot images and all replicates are shown in Figures S4 and S5.

Two-way ANOVAs revealed significant effects of *sleep* for collagen (*p* = 0.01), TGF-β (*p* = 0.04), and p-AKT (*p* = 0.001). In contrast, p-ERK and the p-ERK/total ERK ratio showed significant effects of age only (*p* = 0.03 and *p* = 0.02, respectively) in two-way ANOVAs. Post hoc analyses indicate that collagen type 1 levels were higher with Sleep Dis (significantly so in Mature Sleep Dis rats, compared to Mature Normal Sleep rats, *p* = 0.04, Figure 12B). TGF-β levels were higher in Young Adult Sleep Dis rats, compared to Young Adult Normal Sleep rats (*p* = 0.01, Figure 12C). Phosphorylated (p)-AKT levels were higher with Sleep Dis rats in general (*p* = 0.004 young adult, *p* = 0.04 mature, Figure 12D) as was the ratio of p-AKT to total AKT (*p* = 0.007 each, Figure 12E and Figure S4). No significant post hoc changes were observed for p-ERK or p-ERK/total ERK ratio (Figure 12F,G and Figure S5).

### 2.13. Pain-Related Behaviors Were Associated with Select Inflammatory and Collagen Changes

Spearman’s rank correlations were used to examine for associations between percent change in pain-related behaviors and tissue outcomes. Forepaw mechanical sensitivity correlated negatively with the number of TRAP-stained cells within nerves (r = −0.40, *p* = 0.02) and collagen deposition (trichome staining) within and around nerves (r = −0.32, *p* = 0.04). The percent change in grip strength correlated negatively with: serum TNF-α levels (r = −0.35, *p* = 0.01), collagen deposition (trichome staining) within and around nerves (r = −0.46 *p* = 0.002), Leder-stained cells within nerves (r = −0.36, *p* = 0.04), collagen deposition (trichome staining) within interossei muscles (r = −0.42, *p* = 0.004), and collagen type I immunoexpression in forelimb flexor muscles (r = −0.39, *p* = 0.046). These findings are shown as graphs in Figure S7.

### 2.14. Several Inflammatory Responses Correlated with Increased Tissue Collagen

Spearman’s rank correlations were used to examine for associations between the various tissue outcomes.

Serum TNF-α levels correlated with immune cells numbers in muscles and nerves, collagen in muscles and nerves, and CTGF in muscles. Specifically, serum TNF-α levels correlated with the following immune cells in muscles and nerves: Leder-stained cells in interosseus muscles (r = 0.42, *p* = 0.03), TRAP-stained cells in interosseus muscles (r = 0.54, *p* = 0.002), CD68+ cells in forearm flexor muscles (r = 0.35, *p* = 0.008), TRAP-stained cells around forepaw nerves (r = 0.35, *p* = 0.04), and toluidine blue stained mast cells in forepaw nerves (r = 0.39, *p* = 0.03). In addition, serum TNF-α levels correlated collagen deposition (trichome staining) within and around nerves (r = 0.44, *p* = 0.004), collagen deposition (trichome staining) in interossei muscles (r = 0.56, *p* = 0.0003), and CTGF immunoexpression in forearm flexor muscles (r = 0.47, *p* = 0.01). These findings are shown as a graph in Figure S8A.

In addition to the correlation with serum TNF-α levels, the collagen deposition (trichome staining) within and around nerves correlated with toluidine blue stained mast cells (r = 0.44, *p* = 0.01), Leder-stained cells (r = 0.33, *p* = 0.047), and TRAP-stained cells (r = 0.51, *p* = 0.003) within and around the nerves. These findings are shown as a graph in Figure S8B.

The collagen deposition (trichome staining) within interossei muscles correlated with serum TNF-α levels, as listed above, as well as with Leder-stained cells in the interosseus muscles (r = 0.54, *p* = 0.0003) (Figure S8C). Lastly, immunoexpression of collagen type I in forearm flexor muscles correlated with numbers of CD68 cells (r = 0.71, *p* = 0.0005), CTGF immunoexpression (r = 0.68, *p* = 0.0005), and TNF-α immunoexpression (r = 0.45 *p* = 0.02), in these same muscles (Figure S8D).

## 3. Discussion

We sought to clarify the effects of chronic and intermittent sleep disturbance on pain-related behaviors, inflammation, and fibrosis in the context of aging on forepaw/forelimb neuro- and myofascial tissues. Based on the literature [6,8,9,10,11,12], we expected that chronic intermittent sleep disturbance would enhance pain behaviors and systemic inflammation. We further hypothesized that the inflammatory would be present in peripheral tissues, and perhaps even fibrosis-related tissue changes, based on research indicating that persistent or chronic inflammation can lead to fibrotic changes in tissues [42,43,44,45]. We observed that 6 weeks of sleep disturbance induced small yet significantly enhanced forepaw mechanical sensitivity and declines in grip strength, as well as low-grade increases in immune cells and collagen in neural- and myofascial tissues, and muscle CTGF in sleep disturbed rats of both age groups, compared to rats with normal sleep. The mature sleep disturbed rats typically showed more immune cells and TNF-α in serum and muscles than the young adult sleep disturbed rats. TGF-β protein levels were significantly higher only in muscles of Young Adult Sleep Disturbed rats. However, collagen type I and p-AKT protein levels were elevated in muscles of sleep disturbed rats, regardless of age. These results suggest that even in uninjured rats that chronic intermittent sleep disturbance can elevate indices of pain, inflammation and fibrosis, or, as potentially for the increase in p-AKT, induce a muscle stress response as discussed further below.

The sleep disturbed rats of both age groups displayed grip strength declines, and mature sleep disturbed rats additionally showed heightened forepaw touch mechanical sensitivity (Figure 2). These results are similar to others showing increased pain behaviors and functional limitations after sleep deprivation or disturbance [1,4,7,8,9,11,12,46,47]. Our observed behavioral declines in these uninjured rats were low-grade, compared to studies examining the effects of sleep disturbance in injured rats [6,35,47,63]. Two studies examining behavioral changes in uninjured healthy rats similarly observed increased mechanical touch sensitivity immediately after a period of rapid eye movement sleep disturbance [30,31]. In another study, rats subjected to sleep deprivation for three days (i.e., acutely) using a disc-on-water method either before or after chronic constriction of the median nerve, followed by 7 days of recovery time, found that the sleep deprivation enhanced nerve injury-induced significant neuropathic pain (mechanical allodynia and thermal hyperalgesia) [35]. However, no increase in pain was observed in uninjured sleep deprived control rats [35]. They postulated that the 7 day recovery time after the imposed sleep deprivation resolved any potential pain behaviors in the sleep deprived control rats [35].

The sleep disturbance used in this study induced several peripheral tissue inflammatory responses (Figure 3, Figure 4, Figure 5, Figure 6, Figure 8 and Figure 10), as expected based on the literature [13,14,15,16,17,18,19,20,21,22,23]. In rodents, acute sleep deprivation for 3 days results in elevated circulating levels of TNF-α and other pro-inflammatory cytokines [34]. In rats with repetitive overuse musculoskeletal injuries, prolonged intermittent sleep disturbance (using the same type of sleep disturbance as used in this study) results in increased serum levels of several pro-inflammatory cytokines [47]. In humans, circulating levels of inflammatory cytokines increase in response to acute or prolonged sleep restrictions [11,19], with each hour of sleep reduction inducing 8% increases in serum TNF-α [19]. The heighted inflammatory responses in the mature sleep disturbed rats (Figure 2, Figure 4, Figure 5, Figure 6, Figure 7 and Figure 10) are similar to findings of others showing that inflammation in response to poor sleep is exacerbated by increases in age [14,25,64,65,66]. Cellular inflammatory responses are also known to occur with sleep disturbance, although to our knowledge, only increases in spontaneous production and release of inflammatory cytokines from circulating mononuclear cells (such as monocytes, macrophages and neutrophils) have been reported before now [16,32,38]. Many of the circulating mononuclear cell elevations in inflammatory cytokines were in response to sleep disturbance after viral and bacterial exposure [14], or with aging [14,16,25], the latter findings supported by our results.

There were no significant changes in corticosterone levels from this sleep disturbance method using chronic intermittent stimulation methods, as previously published when using this same method [6,39]. Serum corticosterone levels do not always elevate with lower stress exploratory wakefulness methods of sleep disturbance [67,68,69]. Although we report corticosterone levels for only one time point in this study (thus missing any prior changes), we did not see increases in serum corticosterone levels in our prior longitudinal analyses of this hormone in response to this type of sleep disturbance [6,39].

We novelly observed that 6 weeks of sleep disturbance in uninjured rats of both age groups resulted in increased collagen deposition and production within and around forepaw nerves and in forelimb muscles of uninjured rats, compared to normal sleep rats of either age group. The increase in collagen deposition in muscles was localized to the endomysium, a normally thin fascial layer located between individual myofibers. It was hard to confirm whether there was also increased collagen deposition in the interossei perimysium since these muscles are bipennate in nature (i.e., attach to a paired adjacent muscle via a central tendon [70] with strong perimysium fascial slips attaching interossei to each other and to the carpal bones). It is interesting that the tissue collagen responses correlated with many of the inflammatory responses (Figure S8). Yet, causality cannot be inferred from the present study in which an anti-inflammatory drug was not utilized.

Our various protein assays revealed several sleep-induced changes in protein synthesis in forearm muscles. Protein expression of collagen type I (using immunohistochemical and slot blot detection methods) was increased in both young adult and mature sleep disturbed rats, similar to the histochemical results of increased collagen staining in forepaw muscles. The collagen type I increases were accompanied by increased CTGF in both young adult and mature sleep disturbed rats, and increased TGF-β in young adult sleep disturbed rats. Both TGF-β and CTGF are upstream inducers of collagen [71,72]. Yet, exactly how muscle protein signaling pathways interact with muscle metabolic changes induced by insufficient sleep is still under investigation [73,74]. One study performed a gene set enrichment analysis on muscle biopsies collected from healthy young adult human males exposed to 5 nights of sleep restriction versus normal sleep [75]. They found increased enrichment of inflammatory and immune-response-related pathways in the sleep restriction group, as well as enrichment of genes related to extracellular matrix reorganization. Such an enrichment of genes related to extracellular matrix reorganization could contribute to increased deposition of matrix proteins (e.g., collagen and CTGF) into the extracellular tissues, as observed in our study.

The observed increase in p-AKT and p-AKT/Total (pan AKT) in the flexor muscles in this current study (Figure 12) is interesting. AKT (Protein Kinase B) is involved in the regulation of protein synthesis in skeletal muscle via the AKT-mTOR (serine/threonine kinase mechanistic/mammalian target of rapamycin) signaling pathways [76], and phosphorylated AKT is a central node of many signaling pathways [77]. However, little is known about how sleep restriction regulates or alters the AKT or mTOR pathways. One study examined the effects of short-term sleep restriction in healthy young males [74]. After two nights of controlled baseline sleep, they were exposed to either five nights of sleep restriction, sleep restriction combined with exercise, or normal sleep [74]. Myofibrillar protein synthesis was lower in the sleep restriction group, but no changes in muscle levels of p-AKT or p-mTOR were observed [74]. In another study examining the effects of one night of total sleep disturbance in seven males and six females), muscle protein synthesis was reduced (primarily in the males) while plasma cortisol levels were increased [78]. Muscles of rats exposed to sleep disturbance for 96 h showed no increase in mTOR activity (and increased corticosterone levels)—only a combination of resistance training and sleep disturbance resulted in increased mTOR activity [79]. Previous studies have shown that a catabolic insult, e.g., denervation of muscles, can increase phosphorylated mTOR levels (indicative of increased muscle synthesis) [80]. An increase in phosphorylated AKT in response to the chronic intermittent sleep disturbance in this current study may be in response to the activated immune system since increased p-AKT has been associated with chronic low-grade inflammatory responses [81,82,83]. Perhaps increased p-AKT expression is a compensatory stress response to promote cell survival [73,84]. More studies are needed to understand the role of p-AKT in chronic sleep disturbance.

An association between sleep and skeletal muscle sarcopenia has been reported [73,85,86], such as after 96 h (i.e., acute) of complete sleep deprivation in rats [79]. We did not observe loss of muscle in either the forepaw interossei muscles or the forearm flexor digitorum muscles. Contributions to sarcopenia are multifactorial and include advanced age, rapid loss of body weight, muscle type, and completeness of the sleep disturbance [73,78,79,86,87]. We utilized young adult and mature (3 or 9–10 mo. of age at onset, respectively), rather than rats that were 12 mo. of age (as used in [87]) or geriatric (~2 years of age or older) in which greater age is associated with more sarcopenia and slower recovery times [86,87]. No significant loss of body weight was observed in the rats in this study, in contrast to studies examining the effects of acute complete sleep deprivation, in which rapid loss of body weight and marked sarcopenia are induced [78,79]. Insufficient sleep has a profound effect on glycolytic (i.e., fast twitch) muscles, but less so in oxidative slow twitch muscles [73,88]. The rat interossei muscles that we examined are composed of slow twitch Type I and intermediate type IIa fibers [89], and rat forearm flexor muscles are a mix of slow and fast types of muscles [90]. We also used intermittent rather than acute complete sleep deprivation. Thus, several study differences could contribute to the lack of muscle loss in the rats of this study.

This study has several limitations. We examined only female uninjured rats, primarily because these rats were generated as controls for other studies examining the interactive effects of sleep disturbance and injury. This limitation is somewhat mitigated by findings that sex is a factor in sleep fragmentation, with females showing higher subsequent elevations than males in inflammatory cytokines [13,18,32]. We collected the tissues only at the 6-week end point. It is quite possible that different proteins were simulated at more acute time points. We could not perform immunohistochemical assays on forepaw tissues due to the long decalcification and paraffin embedding process necessary for their examination, as discussed previously in the literature [91]. We mitigated this by collecting and examining the adjacent flexor digitorum muscles that are also involved in forearm functions. We cannot address the effects our perturbations on sleep architecture or pattern since we did not perform electroencephalogram assays in our efforts to induce sleep disturbance with as little stress induction as possible, and in an effort to maintain the rats as uninjured (rat electroencephalogram assays involve anesthesia and surgical placement of instrumentation) (which are strengths). Lastly, we did not examine for any potential central nervous system changes since that was beyond the scope of this study. Examination of central nervous system changes is being pursued in another study.

Thus, we found low-grade yet significant sensorimotor behavioral declines, and inflammatory and fibrosis-related tissue responses in neuro- and myofascial tissues after 6 weeks of intermittent sleep disturbance in uninjured rats, with mature rats showing the greatest immune responses. Future studies will examine whether injury enhances these inflammatory and fibrosis-related tissue responses.

## 4. Materials and Methods

### 4.1. Overview of Animals

All experiments were pre-approved by the Institutional Animal Care and Use Committee (protocol # 5054, approved 25 March 2021 through present time) and were compliant with NIH guidelines for the humane care and use of laboratory animals [92]. Sex is an important factor in the modulation of pain [93,94]; the inclusion of males would have introduced a confounding factor into this study with four groups of female rats (Figure 1A).

Rats were housed in an AAALAC-accredited central animal facility under 12 h light: 12 h dark cycle conditions, at 22–23 °C room temperatures. They were housed in standard filter topped cages that were ventilated and with hardwood chip rodent bedding that was changed twice per week. They were provided free access to food and water. Rats were acclimated to the animal facility for 1 week and maintained in group housing (2 per cage) until experimental onset. Thereafter, they were housed individually (necessary to assess sleep and/or to disturb sleep in home cages [6,53]). All rats were handled five days/week and provided cage enrichment toys daily in their home cages (tunnels and chew toys).

Young adult (3 mo. of age at onset of experiments) or mature adult (9–10 mo. of age at onset) uninjured female rats were exposed to either normal sleep or disturbed sleep for 9 h per day on 4 random days/week for 6 weeks (Figure 1A). For this, 65 Sprague-Dawley rats were procured (Charles River, Wilmington, MA, USA): 32 young adult (2.5 mo. of age) and 33 mature female (8–9 mo. of age). All rats were aged in-house until they reached 3 or 9–10 mo. of age. Rats were checked daily for health, food and water by laboratory or institutional laboratory animal staff, under the supervision of an institutional veterinarian. Rats were weighed weekly. Two young adult rats were excluded during the study due to presence of a tumor or thoracic cardiovascular malformation. Four mature rats were excluded due to the development of lethargy from unknown reasons (n = 1), hind paw ulceration (n = 1), development of an eye problem (n = 1), or death from unknown reasons (n = 1 prior to any testing or treatment), leaving 59 total rats by study end. These were divided into four groups: (1) young adult rats exposed to normal sleep, n = 17; 2) young adult rats exposed to disturbed sleep, n = 13; 3) mature rats exposed to normal sleep, n = 11; and 4) mature rats exposed to disturbed sleep, n = 18 (Figure 1A).

### 4.2. Sleep Disturbance (Provided for 6 Weeks)

Sleep disturbance was performed using environmental stimulation, as previously described (see Appendix A Methods in [6]). This was provided during the rats’ sleep phase (9 h from 07:00 to 16:00, as rats are nocturnal) on four random days of the week to avoid circadian and sleep-pattern adaptations. Further explanation of this method is provided in the Supplemental Files of this manuscript.

### 4.3. Behavioral Assays

All animals were acclimated to assay apparatuses and experimenters, with at least 30 min of acclimation to each assay apparatus, prior to any data collection. Attempts were made to keep testers naïve to group assignment. Forepaw sensitivity to mechanical touch stimulation was assessed bilaterally at 6 weeks, using previously described methods [6]. The number of withdrawal responses to 10 probings each of monofilaments that bend at 0.16, 0.4, 1, or 4 cN (North Coast Medical, Inc., Morgan Hill, CA, USA). Right and left limb data were averaged and reported. Reflexive grip strength was used as a measure of muscle myalgia and was performed as previously described [95], at baseline and after 6 weeks of normal sleep or disturbed sleep. Right and left limb grip strength data was averaged, and are reported as the percent change from baseline for each rat, as previously described [95].

### 4.4. Tissue Collection

On the day of euthanasia, animals were deeply anesthetized with 5% isoflurane in oxygen and then euthanized by performing thoracotomy and cardiac puncture. Blood was drawn at this point for later serum biomarker analysis. The flexor digitorum muscle from one forelimb per rat was collected, rinsed in sterile saline, and flash frozen for later biochemical assays. The forepaw of this same limb was removed and immersion fixed in buffered 4% paraformaldehyde for 48 h, for later paraffin embedding and histochemistry. Thereafter, animals were transcardially perfused with buffered 4% paraformaldehyde, as previously described [52]. After perfusion fixation, forepaw and forelimb tissues of the other limb were collected, immersion fixed in buffered 4% paraformaldehyde for 48 h before further tissue preparation.

### 4.5. Serum ELISA

Collected blood was centrifuged at 12000 revolutions per minute at 4 °C for 20 min. Serum was collected, aliquoted and stored at −80 °C, until assayed for levels of tumor necrosis factor alpha (TNF-α) and corticosterone using commercially available ELISA kits (anti-TNF-α, EA100366, OriGene, Rockville, MD, USA, with a sensitivity of 1 pg/mL) and mouse/rat corticosterone (55-CORMS-E01, Alpco, Salem, NH, USA, with a sensitivity of 6.1 ng/mL).

### 4.6. Tissue Processing for Histochemistry and Immunohistochemistry

The fixed forepaws were decalcified and paraffin embedded, as previously described [52]. They were sectioned into 5 micron thick longitudinal sections that were placed onto charged slides (Fisherbrand™ Superfrost™ Plus Microscope Slides, 12-550-15, Thermo Fisher Scientific, Waltham, MA, USA). Enough sections were prepared to allow for at least 3 replicates per stain, per side, and per animal.

Subsets of sections underwent: (1) Masson’s trichrome staining (in which collagen stains blue and muscle red); (2) 0.1% toluidine blue with Eosin Y counterstaining (detects mast cells a red-purple color) (metachromatic staining); (3) Leder staining (detects both mast cells and neutrophils as a blue black color) [96]); (4) Hematoxylin and eosin (H&E); and tartrate-resistant acid phosphatase (TRAP) staining (detects hemopoietic cells producing this hydrolytic enzyme, including mononucleated macrophages [97]), using Leukocyte Acid Phosphatase (TRAP) Kit (387A-1KT, Millipore Sigma, Burlington, MA, USA) with minor modifications. These modifications were, in brief, after deparaffinization slides were incubated in Tris-HCl buffer (PH 9.0) for 1 h at 37 °C following the incubation for 2 h in 37 °C in Fast Garnet GBC-Naphthol AS-Bl Phosphate-Tartrate solution, followed by and counterstaining with Hematoxylin (Gill 2, 22-050-201, Epredia, Fisher Scientific). Brightfield photographs were acquired on a Nikon Eclipse E800 microscope (Nikon, Melville, NY, USA) that was linked to a digital camera (Gryphax Jenoptik, Jena, Germany).

The fixed forearm flexor muscles were prepared for cryosectioning and immunohistochemistry, as previously described [98]). These cryosections were immunostained in batched sets for collagen type 1, CD68 (a marker of monocytes and macrophages in rats), TNF-α, and CTGF, using previously described methods [52] and antibodies listed in Table 1. Sections were counterstained with DAPI and coverslipped with VETASHIELD Vibrance, Antifade Mounting Medium (Vector Laboratories, Inc., Newark, CA, USA). The specificity of these antibodies has been previously determined [99].

### 4.7. Quantification of Stained Sections

Quantification of histochemical or protein immunoexpression was performed as previously described [52,100]. Three fields were quantified per muscle and per rat in a blinded fashion.

In forepaw tissues, numbers of toluidine blue stained mast cells and Leder-stained cells were quantified within nerve profiles. TRAP-positive mononucleated cells and relevant amount of collagen were quantified within and around nerve profiles, as previously described [100]. In intrinsic forepaw muscles (with a focus on interossei muscles), toluidine blue stained mast cells, TRAP-positive cells, Leder-stained cells, relevant amount of collagen and relative amount of muscle (stained red after Masson’s trichrome staining) were quantified, as previously described [52,100]. Forepaw tendons were examined for histopathological changes in H&E stained sections, using previously described scoring methods [101,102]. In immunostained sections, the percent area with collagen type 1, TNF-α and CTGF immunostaining, and numbers of CD68+ cells were quantified in the mid- to proximal portion of the crossectionally cut flexor muscles, using previously described methods [100]. In addition, the greatest cross-sectional area (CSA) of each flexor digitorum muscle was quantified in at least three cross-sections per muscle.

### 4.8. Slot Blots and Western Blots

The unfixed flexor digitorum muscles that were collected for biochemistry were rinsed in sterile saline and flash frozen before storage at −80 °C until use, at which time they were thawed on ice and homogenized in sterile, ice-cold, phosphate-buffered saline (PBS) containing fresh proteinase inhibitors (1 tablet per 25 mL of PBS; cOmplete EDTA free Protease Inhibitor tablets, 5056489001, Sigma-Aldrich, Inc., St. Louis, MO, USA). Muscle samples were homogenized, separately, in 300 to 500 mL of the buffer using porcelain mortars and pestles. Homogenates were centrifuged at 12,000 rpm for 15 min at 4 °C, and supernatants collected. After determining protein content in the samples using a bicinchoninic acid (3225, BCA Protein assay, Pierce^TM^, Thermo Fisher Scientific, Waltham, MA, USA), supernatants were aliquoted and stored at −80 °C until assayed for select proteins using slot blot or Western blot methods.

A slot blot microfiltration device was used (Bio-Dot^®^ SF Apparatus, 1706542, Biorad, Hercules, CA, USA), per manufacturer’s directions and described methods [103]. After transfer, membranes were blocked in 5% BSA in tris buffered saline (TBS) for 1 h and then incubated overnight at 4 °C with primary antibodies described in Table 2, diluted 1:1000 in 5% BSA in TBS with Tween-20 (TBST). After washing the membrane, they were incubated with appropriate secondary antibodies from Li-Cor (IRDye^®^ Infrared Dyes, LI-COR, Lincoln, NE, USA) diluted 1:20,000 in TBST with 5% BSA, for 1 to 1.5 h. Immunostained membranes were imaged on a Licor Odyssey CLx (9140, Lincoln, NE, USA). Image J (version 1.54j, Bethesda, MA, USA) was used to evaluate immunostained and Ponceau-S stained band density, as previously described [103]. Bands of p-AKT and total (pan) AKT as well as p-ERK and total ERK, were compared as a ratio (p-AKT/total AKT, and p-ERK/total ERK). Slot blots were repeated until 5–8 different samples per group were assayed, in duplicate.

Antibody specificity for the collagen, TGF-β, p-ERK and total ERK antibodies have been previously determined by our lab [98,99]. To determine the specificity of the p-AKT antibody, a pre-cast 4–12% Tris-Glycine gels (Novex^TM^ WedgeWell^TM^ 4–12% Tris-Glycine Gels, Invitrogen) was used. Gels were run as previously described [98] and incubated with the same p-AKT primary antibody described above, diluted 1:1000 overnight at 4 °C with shaking. Membranes were washed in TBS with 0.05% Tween and then incubated for 1 h with an 800-conjugated secondary antibody (IRDye^®^ Infrared Dyes, LI-COR), diluted 1:5000. Images were obtained using a LI-COR System and examined for molecular weight of the detected protein. Figure S6 shows that the p-AKT antibody detected a single band at approximately 60 kDa in the flexor muscles.

### 4.9. Statistical Analysis

The sample size for this study was derived from our previous studies using young adult or mature rats [99,100]. From these data, a power analyses set at conservative thresholds of 80% power and a 0.05 alpha level indicate that at least 5 rats/group were needed for histological and biochemical analyses. In this study, this power analysis was met, with similar or more animals per group: n = 5–12/group used for histological analyses, n = 5–8/group for biochemical analyses, and n = 8–16/group for behavioral analyses, with exact number indicated in the scatter plot bar graphs and figure legends.

GraphPad PRISM version 10.4.2 (GraphPad Software, Boston, MA, USA) was used for the statistical analyses. A 3-way repeated-measures mixed-effects model (REML) was used to assess: body weight using the factors *time*, *age* (young adult versus mature), and *sleep* (normal sleep versus disturbed sleep); and percent change in forepaw mechanical sensitivity at week 6 of the experiment was similarly assessed using the factors, *monofilament size*, *age*, and *sleep*; each was followed by Tukey’s post hoc multiple comparisons tests. Two-way ANOVAs with the factors, *age* and *sleep*, were used to assess: (1) percent change in grip strength; (2) serum TNF-α levels; (3) all histological and immunohistochemical data; and (4) slot blot findings. Each two-way ANOVA was followed by Sidak’s post hoc multiple comparisons tests. *p*-values of <0.05 were considered statistically significant. Data are expressed as mean  ±  standard error of the mean (SEM). Post hoc outcomes are presented in the text, figures and/or tables.

Correlations were performed using Spearman’s rank correlation tests.

## 5. Conclusions

We found low-grade yet significant sensorimotor behavioral declines, and inflammatory and fibrosis-related tissue responses in neuro- and myofascial tissues after 6 weeks of intermittent sleep disturbance in uninjured rats, with mature rats showing the greatest immune responses. Future studies will examine whether injury enhances these inflammatory and fibrosis-related tissue responses.

## Figures and Tables

**Table 1:** Antibodies used for immunohistochemistry.

Antibody Name	Catalog #	Source	Dilution
Anti-collagen type 1	C2456	Sigma-Aldrich, Saint Louis, MO, USA	1:500
Anti-CD68	MCA341	Biorad, Hercules, CA, USA	1:100
Anti-TNF-α	GTX110520	Genetex, Zeeland, MI, USA	1:150
Anti-CTGF	23936-1-AP	Proteintech, Rosemont, IL, USA	1:100
Alexa Fluor 647 Goat anti-mouse IgG	115-605-166	Jackson ImmunoResearch	1:100

**Table 2:** Antibodies used for slot blots.

Antibody Name	Catalog #	Source	Dilution
Anti-collagen type 1	C2456	Sigma-Aldrich	1:1000
Anti-TGF-β (transforming growth factor beta 1, Clone 9016	MAB240	R&D Systems, Minneapolis, MN, USA	1:1000
total ERK (Extracellular signal-regulated kinases, anti-p44/42 MAPK)	total Erk1/2, 137F5	Cell Signaling Technologies, Danvers, MA, USA	1:1000
p-ERK (anti-Phospho-p44/42 MAPK)	pErk1/2; Thr202/Tyr204, D.133.14.4E	Cell Signaling Technologies	1:1000
Anti-phospho-AKT (activated form of the serine/threonine kinase AKT (Protein Kinase B)	Ser473, D9E, 4060S	Cell Signaling Technologies	1:1000
Total (pan)-AKT	40D4, 2920	Cell Signaling Technologies	1:1000

## Data Availability

The raw data for this study is available in the Supplemental Files.

## Supplementary Materials

The following supporting information can be downloaded at: https://www.mdpi.com/article/10.3390/ijms27146106/s1.

## Abbreviations

The following abbreviations are used in this manuscript:

ANOVA	Analysis of variance
CD68	a marker of monocytes and macrophages in rats
cN	centiNewton
CTGF	Connective Tissue Growth Factor
ELISA	Enzyme-Linked Immunosorbent Assay
h	hour
m	muscle
mo.	month
N	nerve
NS	Normal sleep
p-AKT	phospho-Akt (activated form of the serine/threonine kinase Akt; Protein Kinase B)
p-ERK	Phospho-p44/42 MAPK (pErk1/2; Thr202/Tyr204)
PI3	phosphatidylinositol 3
SEM	standard error of the mean
SD	Sleep Disturbed
Sleep Dis	Sleep Disturbed
TGF-β	Transforming Growth Factor be
TNF-α	tumor necrosis factor alpha
TRAP	tartrate-resistant acid phosphatase
