# Supplementary material for: Chronic Sleep Disturbance Enhances Inflammation and Collagen Production in Neural- and Myofascial Tissues in Uninjured Rats
Source: Int J Mol Sci. 2026 Jul 8;27(14):6106. doi: 10.3390/ijms27146106 (PMC13409856; doi:10.3390/ijms27146106)
Supplement: Supplementary file 1 [file ijms-27-06106-s001.zip › ijms-4350708-supplementary.pdf]

Chronic Sleep Disturbance Enhances Inflammation and Collagen Production in Neural- and Myofascial Tissues in Uninjured Rats

Mikhail A. Kolpakov [1], Betsy A. Kalicharan [1], Lewis Bright-Rowe [1], Frank L. Chen [1], Khyleisha A. Caesar [1], Yasmine B. Dahleh [1], Abby Kegg [1], Brendan A. Hilliard [1], Soul M. Moreno [1], Megan Van Der Bas [2], Parth R. Patel [2], Shrey Sitaram [1] and Mary F. Barbe [1,*]

[1] Aging + Cardiovascular Discovery Center, Department of Cardiovascular Sciences, Lewis Katz School of Medicine, Temple University, Philadelphia, PA 19140, USA; mikhail.kolpakov@temple.edu (M.A.K.); frank.chen0001@temple.edu (F.L.C.); yasmine.dahleh@temple.edu (Y.B.D.); brendan.hilliard@temple.edu (B.A.H.); soul.moreno@temple.edu (S.M.M.); shreysitaram@temple.edu (S.S.)

[2] Biomedical Science Graduate Program, Lewis Katz School of Medicine, Temple University, Philadelphia, PA 19140, USA; megan.vanderbas@temple.edu (M.V.D.B.); ppatel2015@temple.edu (P.R.P.)

* Correspondence: mary.barbe@temple.edu; Tel.: +1-215-707-6422

Supplemental Methods for Sleep Disturbance methods and Supplemental Figures 1-8 are shown with explanatory legends.

Supplemental Methods.

Sleep disturbance

Sleep disturbance involved frequent arousing of rats during the light-phase (12 h from 0600-1800, as rats are nocturnal) on four random days of the week to avoid circadian and sleep-pattern adaptations. This was achieved by replacing an old object with a new stimulatory object in their cage when the rat appeared to become drowsy. Stimulatory objects included plastic toys and tubes of different sizes, tube lids, marbles and nesting material. When necessary, additional arousal was achieved by disturbing the bedding/nest and/or introducing objects containing sucrose food reward pellets that can be sensed but not accessed by the animal. Cages were also exchanged twice a day (1200 and 1400) for fresh ones to provide additional stimulation. This method avoids the confounding impact of acute stress associated with current sleep restriction/disturbance methods and better reflects poor sleep in humans in the real world. Traditional models of sleep disturbance and/or deprivation (partial, intermittent or complete) do not relate well to the human experience. Three major issues underlie this problem. First, most models are stress-related, involving exposure to acute stressors (e.g., loud/unpredictable noises, foot shock, grid over water in which animals fall from their confined grid if they fall asleep) (10), which are known to substantial impact the immune system and pain (11-13). Second, other models involve either pharmacological, genetic or invasive interventions, resulting in a host of side-effects unrelated to the normal effects of poor sleep (10). Third, poor sleep is usually induced and studied acutely and/or only over a short period of time (i.e., less than a week) (10). In humans, however, poor sleep is generally of lower intensity and endured over longer periods of time, especially in the context of pain (14, 15). An additional but perhaps less critical issue is the fact that many, if not most, bouts of poor sleep occur and/or worsen in response to interacting with our environment past "normal" bedtime hours. As described above, many current animal models do not account for these important "real-world" factors. Our method used here addresses these issues.

Corticosterone levels do not elevate in serum when using this type of exploratory wakefulness (Owen and Veasey 2020). Toth et al has indicated that perturbations of rodents in their home cages using sensory stimulation or altered environmental stimulations are needed to more match the human condition (Toth and Bhargava 2013).

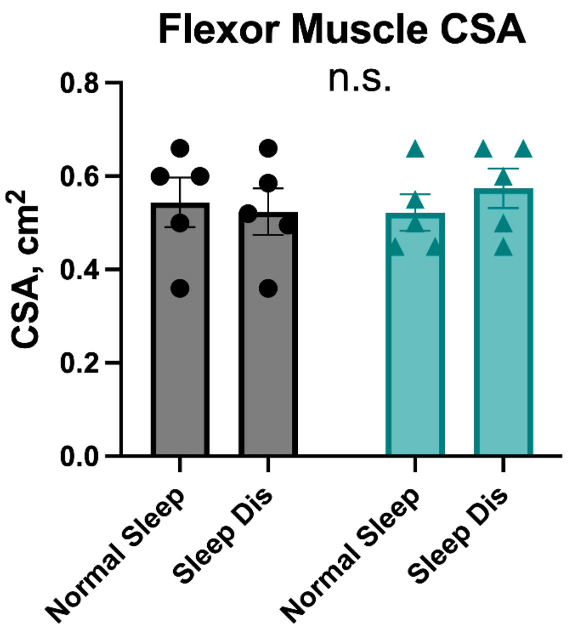

Figure S1. Cross-sectional area (CSA) of the widest part of the forelimb flexor digitorum muscle. No significant (n.s.) differences were observed between the groups. *n*=5/group.

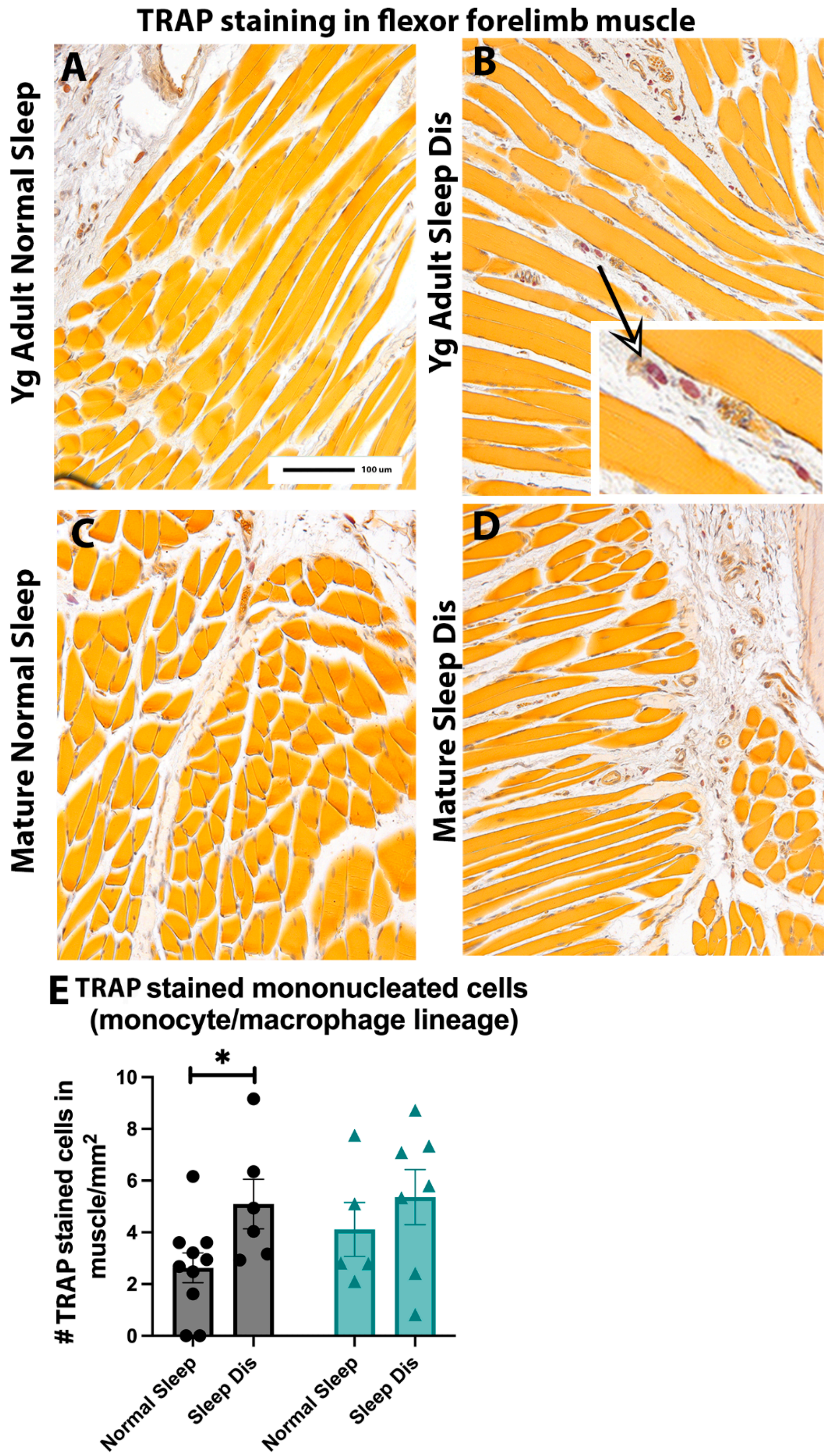

Figure S2. TRAP stained monocyte and macrophage lineage cells in the flexor forelimb muscles. (A-D) TRAP stained monocyte and macrophage lineage cells. Inset in B shows higher power image of the

stained cells (white arrow). (E) Quantification of TRAP stained cells in flexor forelimb muscles and associated fascia. Cells were observed primarily in perimysium fascia (septa) between fascicle bundles. Number (n) of animals per group: Young Adult Normal-Sleep, n = 10; Young Adult Sleep Dis, n = 6; Mature Normal-Sleep, n = 5; and Mature Sleep Dis, n = 7. *$p<0.05$.

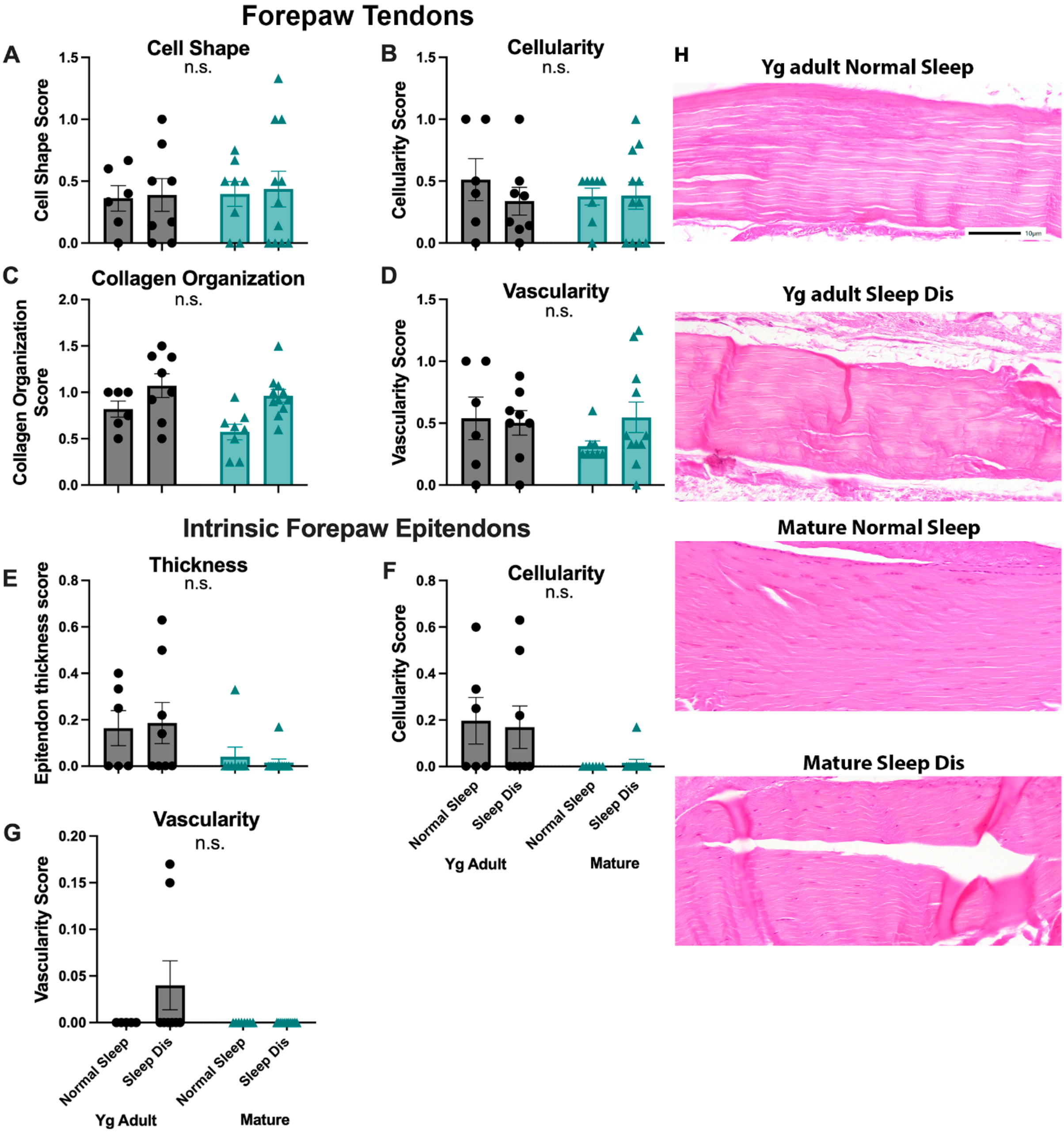

Figure S3. Histological characteristics of intrinsic tendons in the forepaw, scored using a modified Bonar system after hematoxylin and eosin staining. (A-D) Tendon proper, in which cell shape, cellularity, collagen organization and vascularity features were scored. (E-G) Epitenons, in which thickness, cellularity and vascularity features were scored. Number (*n*) of animals per group: Young Adult Normal-Sleep, *n* = 6; Young Adult Sleep Dis, *n* = 8; Mature Normal-Sleep, *n* = 8; and Mature Sleep Dis, *n* = 11. n.s. = not significant. (H) Representative forepaw tendon images from each group, after hematoxylin and eosin staining.

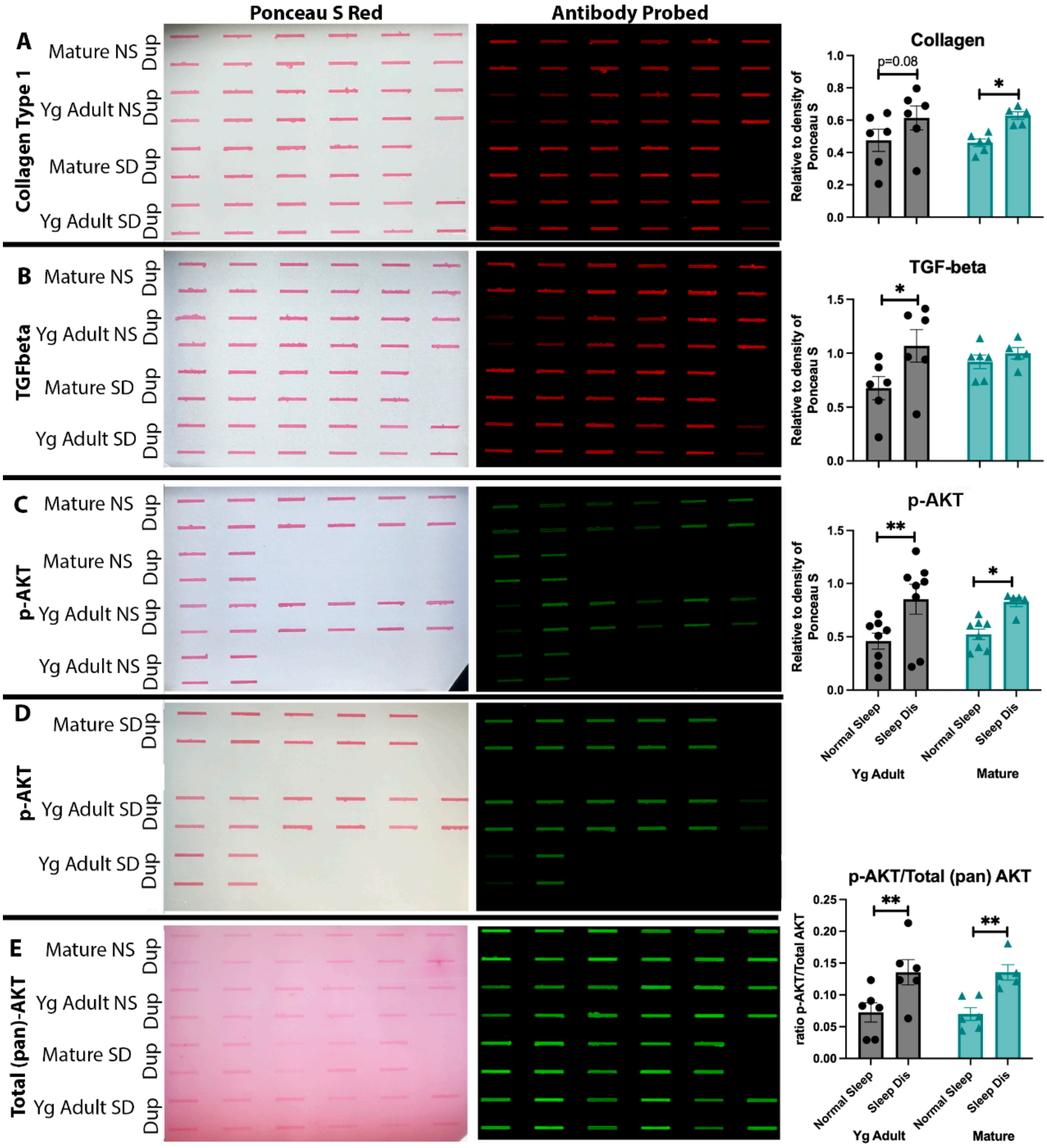

Figure S4. Whole slot blots for examination of collagen type 1, TGF-beta, p-AKT, and total (pan) AKT in homogenized samples of forelimb flexor digitorum muscles via slot blot. For each membrane, Ponceau S staining is shown on the left (stained before antibody-detection steps), and membranes were loaded with 5-8 different samples from the same group along the same row (labeled on the left by age and sleep exposure, with NS = normal sleep, and SD = sleep disturbed). Duplicates of each sample were loaded in column formation. (A and B) After washing out the ponceau S stain, membranes were probed with primary antibodies against collagen type 1 and TGF- beta. (C and D) After washing out the ponceau S stain, both membranes were probed with primary antibodies against p-AKT. (E). After washing out the ponceau S stain, membranes were probed with primary antibodies against total (pan) AKT. Quantification of the density each band relative to the Ponceau S staining of the same band, or p-AKT/total AKT, with *n* = 5-8/group, are shown on the right. *p<0.05 and **p<0.01, compared between groups as shown.

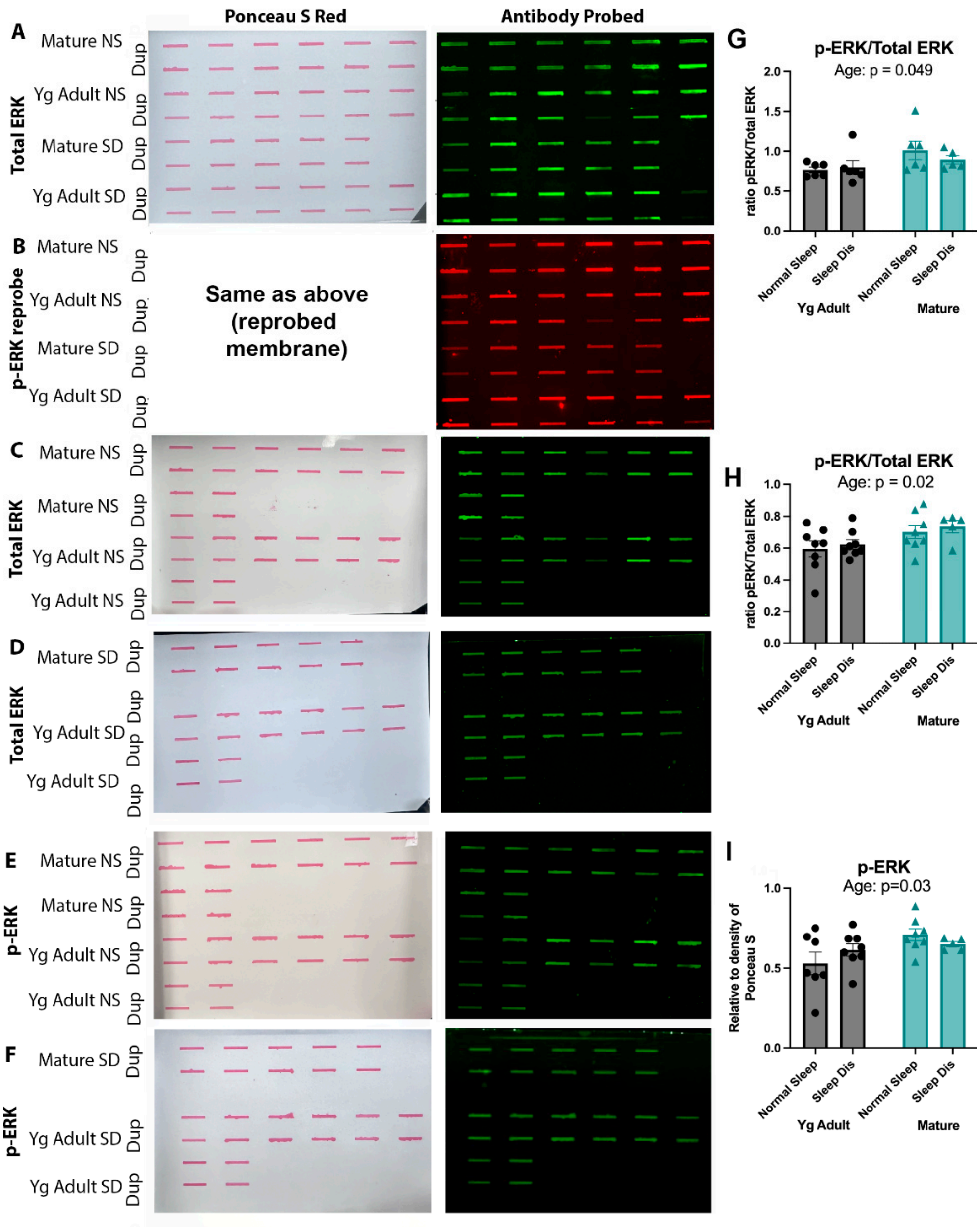

Figure S5. Whole slot blots for examination of total ERK and p-ERK in homogenized samples of forelimb flexor digitorum muscles via slot blot. For each membrane, Ponceau S staining is shown on the left (stained before antibody-detection steps), and membranes were loaded with 5-8 different samples from the same group along the same row (labeled on the left by age and sleep exposure, with NS = normal sleep, and SD = sleep disturbed). (A and B) After washing out the ponceau S stain, this membrane was probed first with a primary antibody against total ERK (panel A), before the same membrane was reprobed with an antibody against p-ERK (panel B) and then reimaged. (C and D) Both membranes were probed for total ERK. (E and F) Both membranes were probed for p-ERK. (G and H). Quantification of the density each p-ERK band relative to the total ERK density from the same band. (I) Quantification of the density each p-ERK band relative to the Ponceau S staining of the same band. *p<0.05 and **p<0.01, compared between groups as shown.

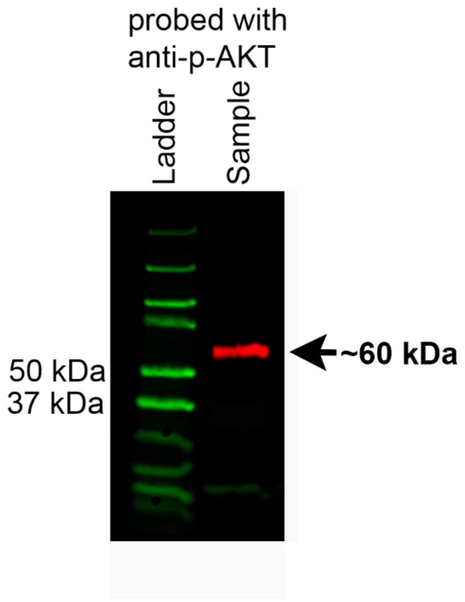

Figure S6. Western blot of a flexor digitorum muscle sample probed with specific antibodies against p-AKT. The p-AKT antibody recognized a single band (red) at approximately 60kDa, the known molecular weight of p-AKT.

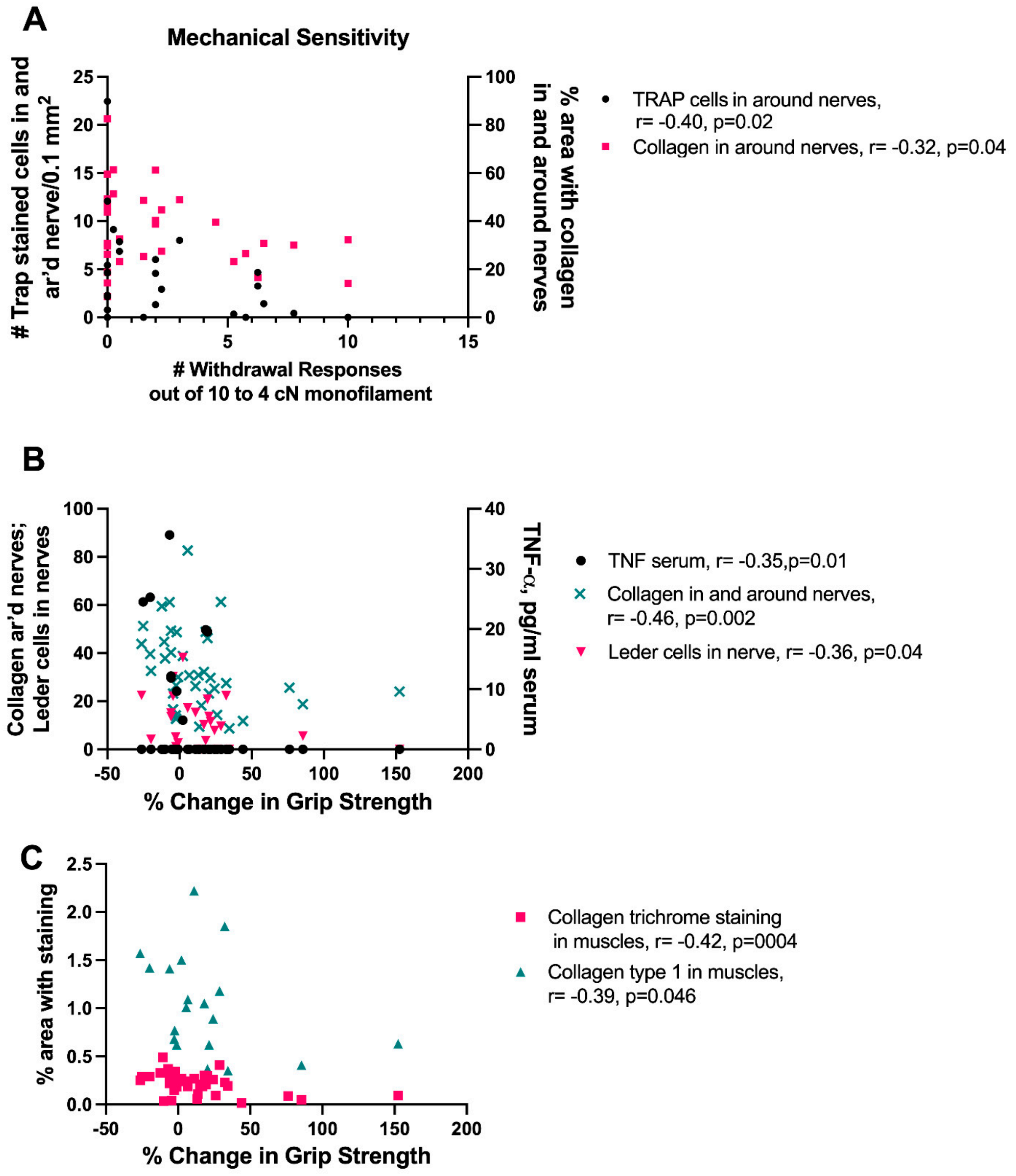

Figure S7. Graphs depicting significant correlations between pain-related behaviors and tissue analytes. (A) Correlations between forepaw mechanical sensitivity to a 4cN monofilament and tissue analytes. (B) Correlations between percent change in grip strength from baseline levels and tissue analytes. Correlation values and p values are reported.

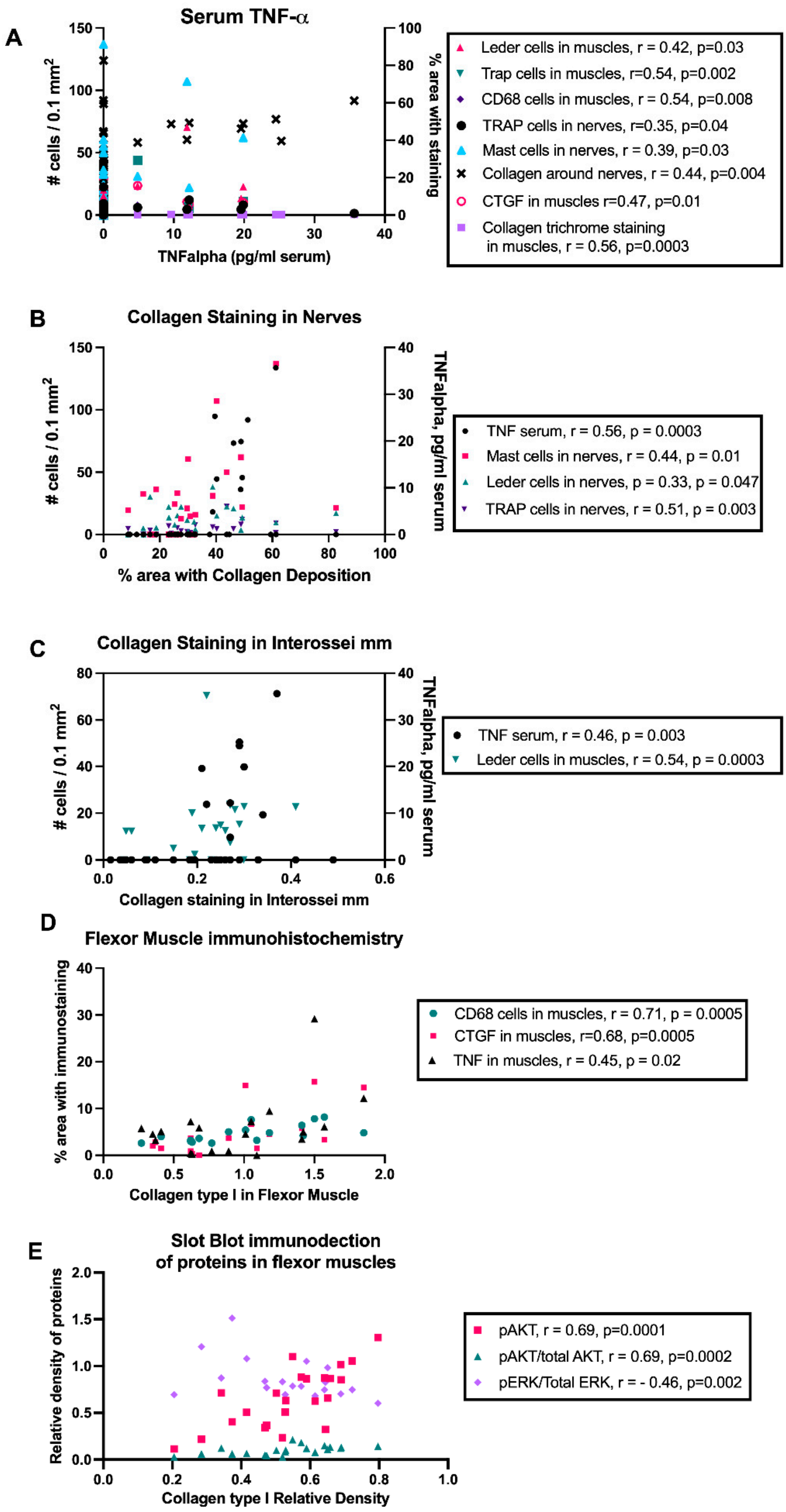

Figure S8. Graphs depicting significant correlations between various tissue responses, e.g., serum TNF-α, neuromuscular inflammatory and/or fibrosis-related responses. (A) Significant correlations with serum TNF-α levels. (B) Significant correlations with percent area with collagen deposition within and around subcutaneous nerves on glabrous surfaces of forepaws (i.e. blue staining after trichrome staining). (C) Significant correlations with percent area with collagen deposition in interosseous muscles of the forepaw (i.e. blue staining after trichrome staining). (D) Significant correlations with percent area with collagen type I immunoexpression in forearm flexor digitorum muscles. (E) Significant correlations with collagen type I protein expression (detected using slot blot) and other proteins examined using slot blot methodology in forearm flexor digitorum muscles. Spearman's rank (r) correlation values and p values are reported.
